# Phylogenetic climatic niche conservatism and evolution of climatic suitability in Neotropical Angraecinae (Vandeae, Orchidaceae) and their closest African relatives

**DOI:** 10.7717/peerj.3328

**Published:** 2017-05-16

**Authors:** Marta Kolanowska, Elżbieta Grochocka, Kamil Konowalik

**Affiliations:** 1 Department of Plant Taxonomy and Nature Conservation, University of Gdańsk, Gdańsk, Poland; 2 Department of Biodiversity Research, Global Change Research Institute AS CR, Brno, Czech Republic; 3 Institute of Biology, Wrocław University of Environmental and Life Sciences, Wrocław, Poland

**Keywords:** Angraecinae, Ecological niche modeling, Orchidaceae, Phylogenetic niche conservatism, Angraecum, Campylocentrum, Dendrophylax

## Abstract

In the present study we investigate the concept of phylogenetic niche conservatism (PNC) within the American species of angraecoid orchids (*Campylocentrum* and *Dendrophylax*) and their closest relatives in the Old World (*Angraecum*) using ecological niche modelling (ENM). The predicted niche occupancy profiles were matched with the outcomes of previous phylogenetic studies to reconstruct the evolution of climatic suitability within the orchid group studied and evaluate the role of niche differentiation in the speciation of Angraecinae. No correlation between preferred niches and taxonomic relationships within the orchid group studied was revealed. The climatic suitability of the majority of the species overlapped each other, either fully or partially. This pattern is also present in the species of other orchid genera. Our research confirms a significant level of PNC in Orchidaceae, even within taxa exhibiting a transatlantic disjunction. The analysis of the evolution of climatic suitability indicated that the adaptation to various climatic conditions is not a factor that has driven speciation within orchids studied.

## Introduction

As defined by Harvey & Pagel (1991), phylogenetic niche conservatism (PNC) is the tendency of lineages to retain their ancestral ecological niche through speciation events. In fact, some of the phylogenetic studies conducted in recent years indicate that major aspects of the niche are more preserved during evolution than expected (Donoghue, 2008; Olalla-Tárraga et al., 2011; Khaliq et al., 2015). However, PNC theory does not suggest that ecological barriers are insurmountable. Obviously, niche evolution occurs. Niche conservatism can be considered as a significant factor in allopatric speciation because it limits adaptation to climatic conditions at the geographic barrier (Wiens & Graham, 2005). Wiens & Donoghue (2004) argue that the interaction between niche conservatism and niche evolution may be critical in the biogeographic history of many groups.

Niche conservatism in species of plants may reflect the opportunities of their ancestors during their diversification. The differences between environmental gradients could be correlated with the palaeo-environmental conditions during the radiation of phylogenetically related lineages. Prinzing et al. (2001) assume that the variation in the characters of species is influenced by their adaptations to their present habitats, but some traits are a legacy from their ancestor. More recent studies (Hadly, Spaeth & Li, 2009) suggest that whilst niche conservatism at high taxonomic levels is primarily driven by inherent life history traits, at the species level it reflects the underlying environmental controls.

PNC is used to reveal the role of ecological divergence in speciation by many authors (e.g., Kozak & Wiens, 2006; Swenson, 2010) and for numerous animal groups (e.g., Cooper, Freckleton & Jetz, 2011; Wellenreuther, Larson & Svensson, 2012; Pearman et al., 2014; Kerr et al., 2015; Rato et al., 2015; Morinière et al., 2016). Relatively little research has so far been conducted on niche conservatism in plants (Serra-Varela et al., 2015; Hawkins, Rodríguez & Weller, 2011; Hawkins et al., 2014; Wasof et al., 2015) and most of these studies focus on the correlation between PNC and patterns in species richness or the composition of assemblages of species. The importance of niche differentiation in the evolution of various flowering plant groups remains poorly recognized.

The great diversity of orchids is most often attributed to their adaptive radiation in response to specific pollinators (e.g., Paulus & Gack, 1990; Cozzolino & Widmer, 2005; Schiestl, 2005), although Otero & Flanagan (2006) suggest that the obligatory orchid–mycorrhizal interactions should be considered as a factor promoting speciation. Gravendeel et al. (2004) argue that the epiphytism rather than pollinator specialization is the reason for the high species richness in orchids. There are only a few studies on PNC and the variation of climatic suitability within the Orchidaceae (Kolanowska et al., 2016) and most of the previous research on this aspect was on invasive species (Kolanowska, 2013; Kolanowska & Konowalik, 2014).

In the present study we combine research on the evolution of climatic suitability in closely related genera with an investigation of PNC in a taxon with a trans-Atlantic disjunction. The group studied, the subtribe Angraecinae Summerh. (Orchidaceae), comprises about 49 genera and exhibits a great variation in form and habit (Carlsward et al., 2006; Micheneau et al., 2008; Pridgeon et al., 2014). Some of the plants in this group produce elongate stems and well-developed leaves, others are characterized by reduced stems and small, scale-like leaves. The greatest diversity of angrecoid orchids is recorded in the Paleotropics, but two genera, *Campylocentrum* Benth. and *Dendrophylax* Rchb. f., occur only in the Americas. The leafless species occur only in the Neotropics (Carlsward, Whitten & Williams, 2003). The evolution of leaflessness apparently has occurred at least twice in the New World (Carlsward et al., 2006) and whilst there are both leafy and leafless species of *Campylocentrum*, *Dendrophylax* includes only leafless species.

The aim of the present study is to provide an insight into the importance of climatic niches in orchid speciation. To investigate PNC within geographically disjunct taxa we evaluated the similarity of the niches occupied by American angraecoid orchids and their closest relatives from Africa. It is hypothesized that species from both continents occupy different niches and that species differentiation within genera is driven by non-climatic factors. However, there are no studies on niche conservatism in this group. To explore the role of adaptation to various climatic conditions in the speciation processes in Angraecinae the evolution of their climatic suitability was reconstructed by combining ecological niche modelling with a phylogenetic analysis.

## Materials & Methods

### Selection of taxa

Only the species included in the phylogenetic studies of Carlsward et al. (2006) and Micheneau et al. (2008) are included in this study. The localities of the populations of the following species are listed: *Angraecum chevalieri* Summerh. *A. cultriforme* Summerh. (=*Angraecoides cultriforme* (Summerh.) Szlach., Mytnik & Grochocka), *A. eichlerianum* Kraenzl. (=*Eichlerangraecum eichlerianum* (Kraenzl.) Szlach., Mytnik & Grochocka), *A. erectum* Summerh. (=*Angraecoides erecta* (Summerh.) Szlach., Mytnik & Grochocka), *Campylocentrum fasciola* (Lindl.) Cogn., *C. lansbergii* (Rchb. f.) Schltr., *C. micranthum* (Lindl.) Rolfe, *C. pachyrrhizum* (Rchb. f.) Rolfe, *C. poeppigii* (Rchb. f.) Rolfe, *C. tyrridion* Garay & Dunst., *Dendrophylax barrettiae* Fawc. & Rendle, *D. fawcetti* Rolfe (=*Polyrrhiza fawcetti* (Rolfe) Cogn.), *D. filiformis* (Griseb.) Benth. *ex* Fawc. (=*Harrisella filiformis* (Sw.) Cogn.), *D. funalis* (Sw.) Fawc., *D. lindenii* (Lindl.) Benth. ex Rolfe (=*Polyradicion lindenii* (Lindl.) Garay), *D. porrectus* (Rchb. f.) Carlsward & Whitten (=*Harrisella porrecta* (Rchb. f.) Fawc. & Rendle), *D. sallei* (Rchb. f.) Benth. ex Rolfe (=*Polyradicion sallei* (Rchb. f.) Garay) and *D. varius* Urb. Based on morphological studies (Kolanowska, Pérez Escobar & Parra Sánchez, 2012; Kolanowska & Szlachetko, 2013; Szlachetko & Kolanowska, 2013a; Szlachetko & Kolanowska, 2013b) we consider *C*. *sullivanii* Fawc. & Rendle as conspecific with *C. fasciola* as these taxa are morphologically indistinguishable. This is also the conclusion of Bogarín & Pupulin (2010).

### List of localities

The occurrence data were obtained from the information recorded with the specimens deposited in herbaria AMES (Orchid Herbarium of Oakes Ames), AMO (Asociación Mexicana de Orquideología), BM (Natural History Museum, London), COL (Universidad Nacional de Colombia), F (Field Museum of Natural History), FLAS (Florida Museum of Natural History), K (Royal Botanic Gardens), MO (Missouri Botanical Garden), NY (New York Botanical Garden), and P (Muséum National d’Histoire Naturelle), as well as from the literature and the original protologues. Herbarium acronyms follow Index Herbariorum (Thiers, 2015). The georeferencing process followed is that used by Hijmans et al. (1999). The geographic coordinates on the herbarium sheet labels were verified. If there were no geographic coordinates on the herbarium label, we used the description of the locality where the plant was collected and assigned coordinates as precisely as possible to this location. The Google Earth (ver. 6.1.0.5001, Google Inc.) application was used to validate all the information gathered. A total of 162 localities that could be precisely located were used in the ENM (5–46 localities per species; Table S1). *Dendrophylax filiformis* (Griseb.) Benth. *ex* Fawc. (= *Harrisella filiformis* (Sw.) Cogn.) and *Dendrophylax funalis* (Sw.) Fawc. were not included in this analysis because of the lack of a precise locality for them.

### Ecological niche modelling and niche similarity

Input data for the ecological niche modelling were 35 bioclimatic variables with a 10 arc minute resolution obtained from the CliMond dataset (Kriticos et al., 2012). To choose an appropriate modelling extent (i.e., the species movement limits described in the M of the BAM diagram; see Barve et al., 2011) terrestrial ecoregions where species occur were selected. Biotic regions serve as a reliable estimate of the area that is accessible to a species and the easiest way to accurately designate the distribution limits of a species (Soberón & Peterson, 2005; Barve et al., 2011). For this purpose, the world map of terrestrial ecoregions was used (Olson et al., 2001). The bioclimatic maps were clipped to include only those regions where it was confirmed that the species occurred. In addition, some island archipelagos and island-like regions were removed from the study area (i.e., Cape Verde archipelago, Ascension Island, Saint Helena, Nile Delta and some smaller features in North Africa north of 19°N). To account for the multicollinearity nature of the initial variables, a principal component analysis (PCA) was performed in R (R Core Team, 2014).

To select an appropriate number of PCA derived maps (i.e., PC axes) the Kaiser-Guttman criterion, which is based on a mean of all eigenvalues was used by selecting only PC axes with eigenvalues larger than this mean. Following this criterion, maps based on the first six principal components were selected (Table S2). The ecological niche modelling was done using a maximum entropy method implemented in Maxent version 3.3.3k (Phillips, Dudík & Schapire, 2004; Phillips, Anderson & Schapire, 2006; Elith et al., 2011), which is based only on species presence. The maximum iterations were set to 10,000 and convergence threshold to 0.00001. Random seeds were used to keep 1,000 bootstrapping runs from using replicate test and training samples. For each run 20% of the data were set aside as test points (Urbina-Cardona & Loyola, 2008). In order to maintain a sufficient sample size for taxa with a small number of occurrences (below 10) duplicate presence records were not removed and presence points were duplicated within a given grid cell. This procedure was only used for taxa that are narrowly distributed insular endemics with a small number of occurrences for which it would be difficult carry out climatic filtering (Varela, Anderson & García-Valdés, 2014). To simplify the interpretation of the probability of a species being present the logistic Maxent output format was chosen.

The model was evaluated using the most common metrics, area under the curve (AUC; Mason & Graham, 2002) and true skill statistic (TSS; Peirce, 1884). AUC was calculated using the Maxent application automatically based on the training localities. Whilst some authors suggest that it may be misleading (Lobo, Jiménez-Valverde & Real, 2008), it seems to be a valid metric for determining the reliability of the fit of the ENM (Warren, Glor & Turelli, 2008). TSS was calculated using maximum training sensitivity plus specificity.

The similarity between the niches occupied by the species studied was measured using Schoener’s D (D; Schoener, 1968) and I statistic (I; Van der Vaart, 1998; Warren, Glor & Turelli, 2008; Warren, Glor & Turelli, 2010) implemented in ENMTools package for R (Warren, 2016), using the methods for calculating environmental distances proposed by Broennimann et al. (2012). Schoener’s D statistic uses direct measures of species density, which in this study are changed to measures of densities of occurrence modelled in environmental space. ‘I’ statistic is based on the modified Hellinger distance that compares two probability distributions. These two metrics range from 0 (no similarity) to 1 (high similarity). To test the importance of distance the niche identity test was used and calculated by the same function that produced the D and I statistics in the ENMTools package for R (Broennimann et al., 2012; Warren, 2016). To visualize the suitable climatic niche of each species, a PCA of the raw climatic conditions occurring at given locations was performed using R (R Core Team, 2014).

Operations on GIS data were carried out on ArcGis 9.3 (ESRI) and R (R Core Team, 2014).

### Phylogenetic analysis

To construct the phylogenetic tree, sequences from ITS and *trn*L-F (Table S3) were aligned using Mafft 6.833b (Katoh & Toh, 2008). Gapcoder (Young & Healy, 2003) was used to code indels. The Alignments were then merged and a Bayesian phylogenetic analysis was performed using MrBayes 3.2.1 (Ronquist et al., 2012). For the nucleotide part, the best model according to the AIC implemented in jModelTest 2.1.1 (Darriba et al., 2012) was used. For the binary coded gaps, a Jukes–Cantor model (Jukes & Cantor, 1969) was used. 15,000,000 generations were performed in two runs, discarding the first 25% as the burning in fraction and sampling every 1,000th tree. To estimate node ages function chronos in package “ape” was used as described previously (Kolanowska et al., 2016). Lambda was set to 20 and ages were estimated using a semi-parametric method based on penalized likelihood where branch lengths indicate mean numbers of substitutions (Sanderson, 2002; Paradis, Claude & Strimmer, 2004). As this calibration indicates splits between *Campylocentrum* and *Dendrophylax* (max. 12.72 Mya, min. 4.64 Mya), and *Angraecum* and *Polystachya* (max. 32.48 Mya, min. 22.2 Mya) we used published divergence times (Givnish et al., 2015; Andriananjamanantsoa et al., 2016).

To reconstruct ancestral climatic suitabilities the Phyloclim package was used (Heibl & Calenge, 2013) which implements the methods originally developed by Evans et al. (2009). Predicted niche occupancy (PNO) was reconstructed and together with the phylogenetic tree were used to infer ancestral climatic suitabilities. PNO integrates species probability distributions (derived using MaxEnt) with respect to climatic variables. Ancestral climatic suitabilities are the PNOs projected onto the phylogenetic tree. They were reconstructed based on the distribution of climatic suitabilities (PNOs) using maximum likelihood and Brownian motion assumption to plot them at each interior node of the tree.

In addition, a Mantel test was used to verify the correlation between the genetic and niche distances (Serra-Varela et al., 2015). This analysis was performed using the Mantel test available in the ADE4 package of R (Dray & Dufour, 2007) and its significance was assessed by performing 9,999 replications. Genetic distances were generated in PAUP* (Swofford, 2002) using the distances predicted by the GTR model and as niche distances Schoener’s D indices were used. GTR distances were chosen based on the best AIC score in the jModelTest 2.1.1 (Darriba et al., 2012).

Age range correlation (ARC, Fitzpatrick & Turelli, 2006) was performed in R using package phyloclim (Heibl & Calenge, 2013). ARC is equivalent to phylogenetic independent contrasts (PIC) and explains how niche similarity (in this case measured using D and I statistics) change over time, which is represented by the nodes of the phylogenetic tree (Fitzpatrick & Turelli, 2006). Monte Carlo resampling with 3,000 replicates was used to assess the statistical significance. This test was used to verify whether any of the observed differences could be explained by phylogeny or whether these are more probably a result of “ecological drift” than “ecological specialization”.

## Results

### Phylogenetic relationships

**Figure 1:**
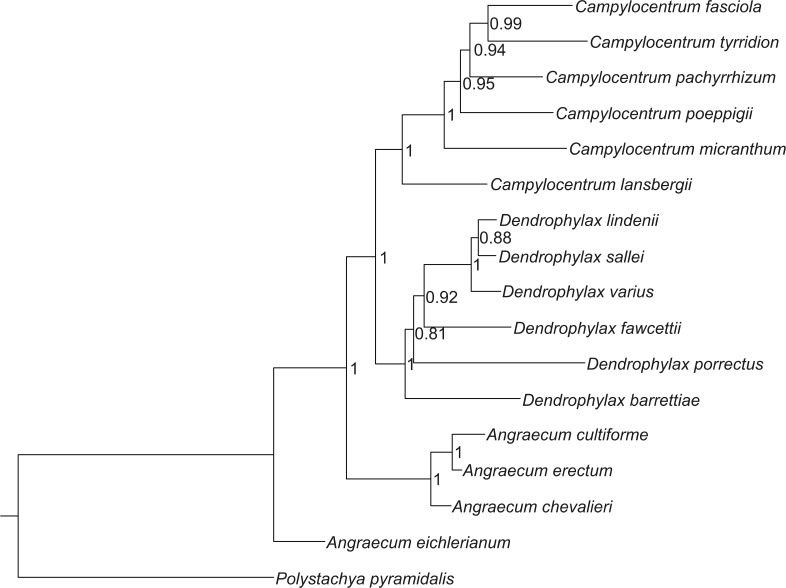
Bayesian inference tree of the combined nuclear ITS and chloroplast *trn*L-F intergenic spacers for 16 species of Angraecinae and one outgroup species. Numbers beside the nodes indicate posterior probability.

The Bayesian tree, based on sequences of both ITS and *trn*L-F, indicate that *Campylocentrum* and *Dendrophylax* form well-supported clades, which originated from African representatives of *Angraecum* (Fig. 1). These analyses support the results of the previous molecular studies of Carlsward et al. (2006), Micheneau et al. (2008) and Szlachetko et al. (2013).

### Evaluation of the modelling of the ecological niche

All projected niche models had high AUC scores ranging from 0.690 to 0.990 (Table 1) indicating the model is good in distinguishing presence data from background data (Phillips, Anderson & Schapire, 2006). The TSS scores showed the same trend but were lower than the AUC scores and ranged from 0.499 to 0.974. The low scores of AUC and TSS are mainly for taxa that are narrow endemics (e.g., those occurring on Caribbean islands). As these scores are close to that of a random prediction they should be treated with caution and may indicate that climate is not a significant factor influencing their distribution (most probably they originated by allopatric speciation, which is not necessarily reinforcing climatic differences).

**Table 1:** The average training AUC and TSS for the replicate runs.

Species	AUC	TSS
*A. chevalieri*	0.972	0.879
*A. cultriforme*	0.767	0.564
*A. eichlerianum*	0.952	0.795
*A. erectum*	0.990	0.937
*C. fasciola*	0.958	0.891
*C. lansbergii*	0.985	0.974
*C. micranthum*	0.886	0.743
*C. pachyrrhizum*	0.940	0.879
*C. poeppigii*	0.934	0.848
*C. tyrridion*	0.914	0.770
*D. barrettiae*	0.918	0.831
*D. fawcetti*	0.975	0.907
*D. lindenii*	0.836	0.773
*D. porrectus*	0.690	0.499
*D. sallei*	0.846	0.736
*D. varius*	0.805	0.735

### Distribution of suitable niches and niche similarity

**Figure 2:**
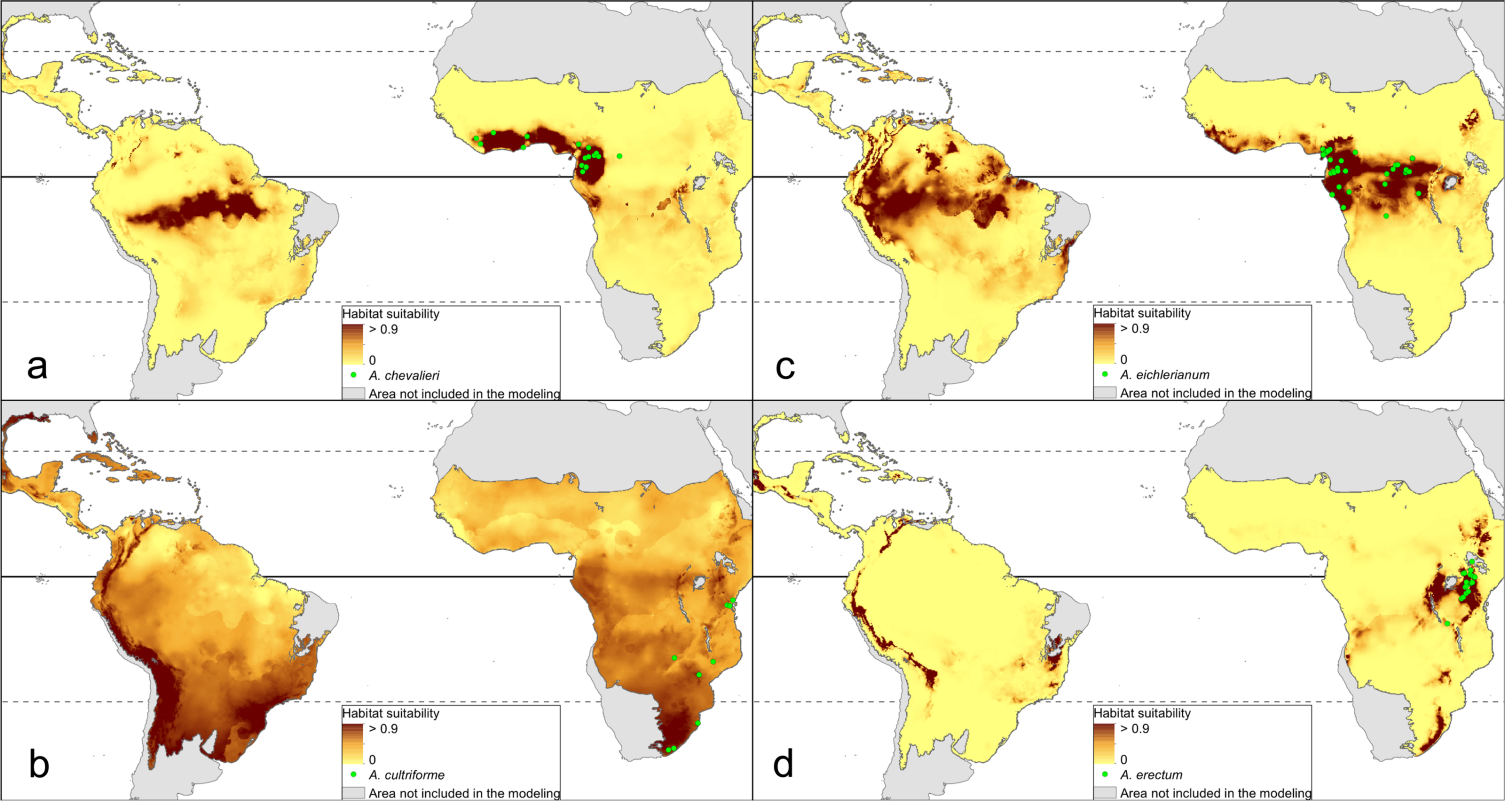
Distribution of suitable niches of *Angraecum* representatives. (A) *Angraecum chevalieri*; (B) *Angraecum cultriforme*; (C) *Angraecum eichlerianum*; (D) *Angraecum erectum.* Localities used in the ENM analysis marked as green spots.

**Figure 3:**
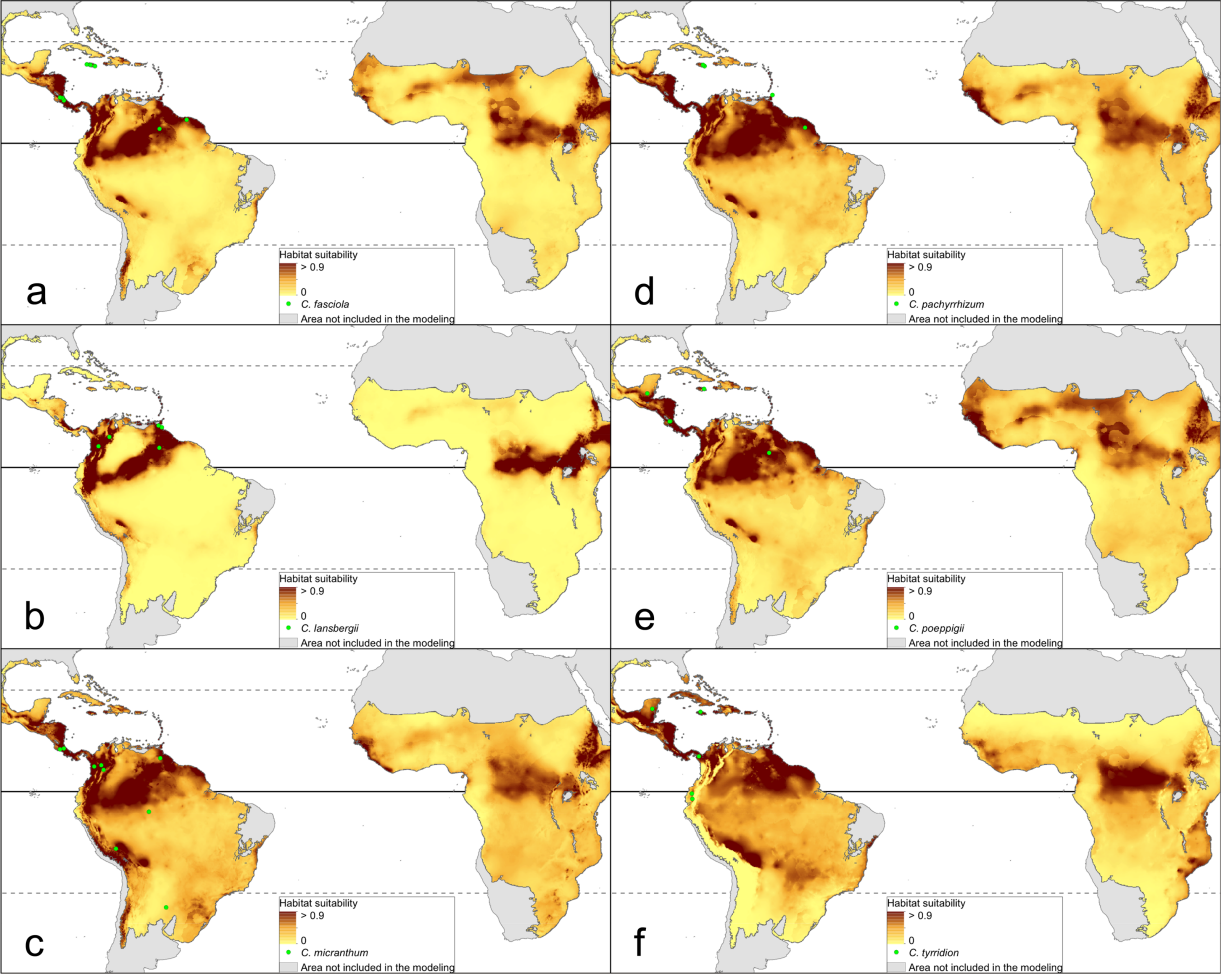
Distribution of suitable niches of *Campylocentrum* representatives. (A) *Campylocentrum fasciola*; (B) *Campylocentrum lansbergii*; (C) *Campylocentrum micranthum*; (D) *Campylocentrum pachyrrhizum*; (E) *Campylocentrum poeppigii*; (F) *Campylocentrum tyrridion*. Localities used in the ENM analysis marked as green spots.

**Figure 4:**
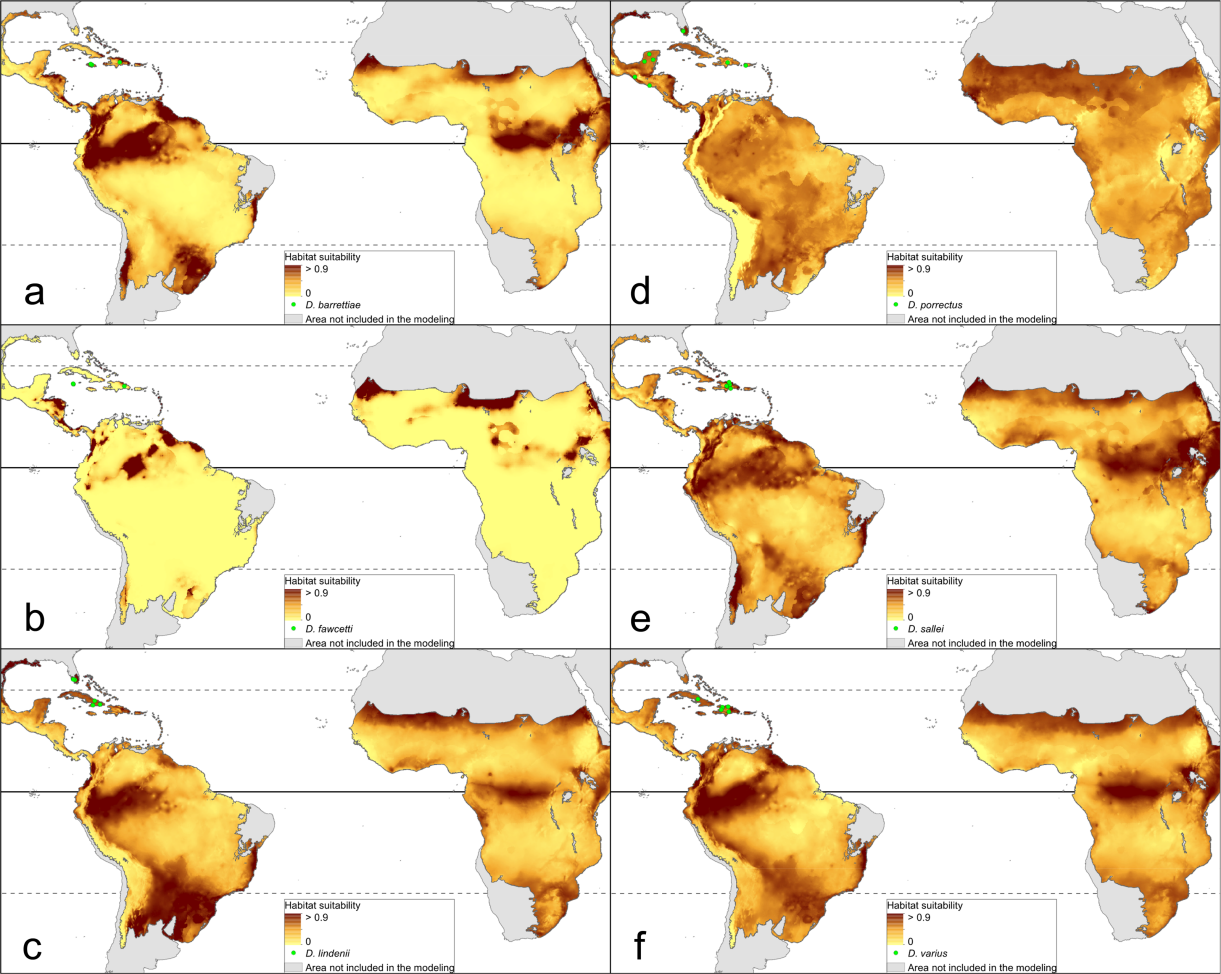
Distribution of suitable niches of *Dendrophylax* representatives. (A) *Dendrophylax barrettiae*; (B) *Dendrophylax fawcettii*; (C) *Dendrophylax lindenii*; (D) *Dendrophylax porrectus*; (E) *Dendrophylax sallei*; (F) *Dendrophylax varius*. Localities used in the ENM analysis marked as green spots.

The distribution of the suitable niches of all species studied is presented in Figs. 2–4. This indicates there is a relatively low cover of suitable habitats for *Dendrophylax fawcettii* and *Campylocentrum lansbergii.*

**Figure 5:**
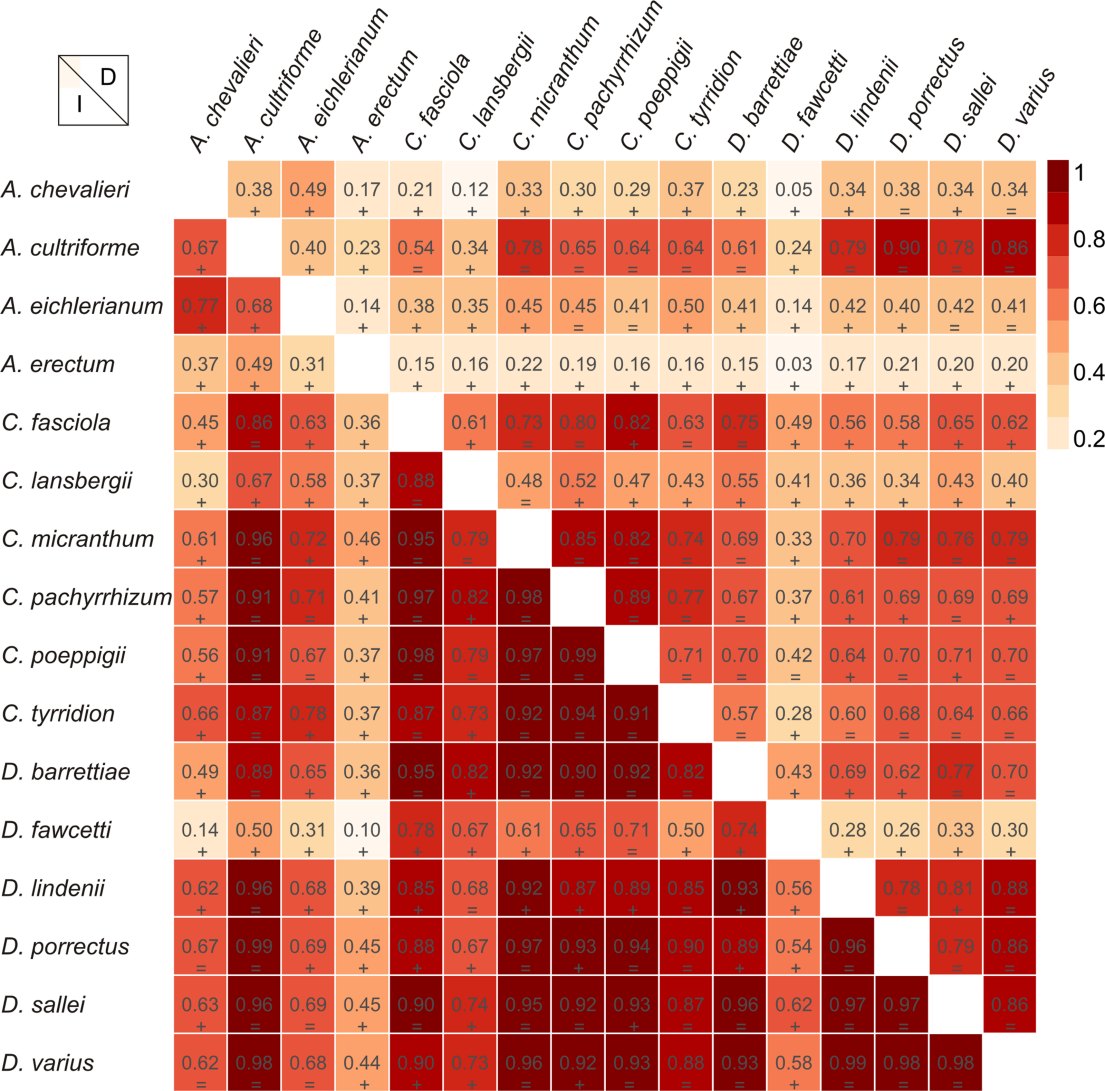
Niche similarities of the studied taxa based on the Schoener’s D (upper triangular) and I statistic (lower triangular). Higher indexes indicate more similarity as shown by the red intensity. Results of the niche identity test are marked below each value where “+^′′^ means that the niches are different (*p* = 0.01) and “=^′′^ indicate the the niches are similar (*p* > 0.01).

**Figure 6:**
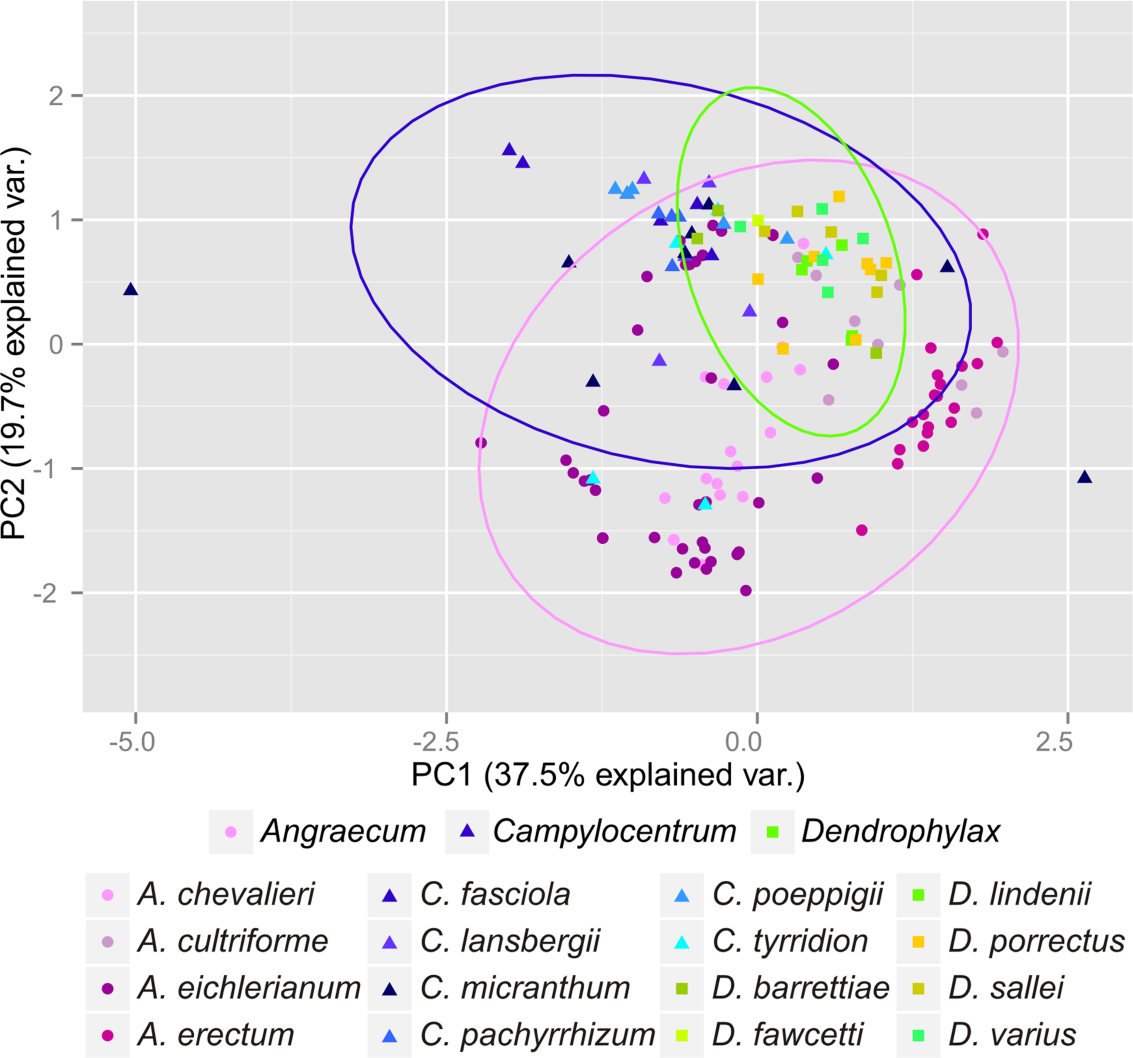
Principal Component Analysis of climatic variables present in the studied populations.

The Mantel test did not reveal a correlation between genetic and environmental distance (*r* =  − 0.14, *p* = 0.87) supporting the hypothesis that niche evolution (i.e., niche difference) is not related to phylogeny and that phylogenetically distant taxa can occupy similar niches. In addition, the results of this analysis (Fig. 5) did not indicate a correlation between the similarity of the niches occupied by the species studied and their phylogenetic relationships (Fig. 1). Overall, the most similar niches are occupied by *Angraecum cultriforme* and *Dendrophylax porrectus* (*I* = 0.99, *D* = 0.90), whilst the greatest differences were recorded for *Dendrophylax fawcettii* and *Angraecum erectum* (*I* = 0.10, *D* = 0.03). The general similarity of the niches occupied by Angraecinae is also visible in the PCA graph (Fig. 6). In this diagram the suitable niches of many clades overlap significantly. It is noteworthy that the niches of all *Dendrophylax* species fall within those of *Angraecum* and *Campylocentrum*. The climatic suitabilities of these particular species also overlap each other and there are no clear boundaries between the genera (taxa) studied in terms of their preferred climatic conditions.

### Ancestral climatic suitability

**Figure 7:**
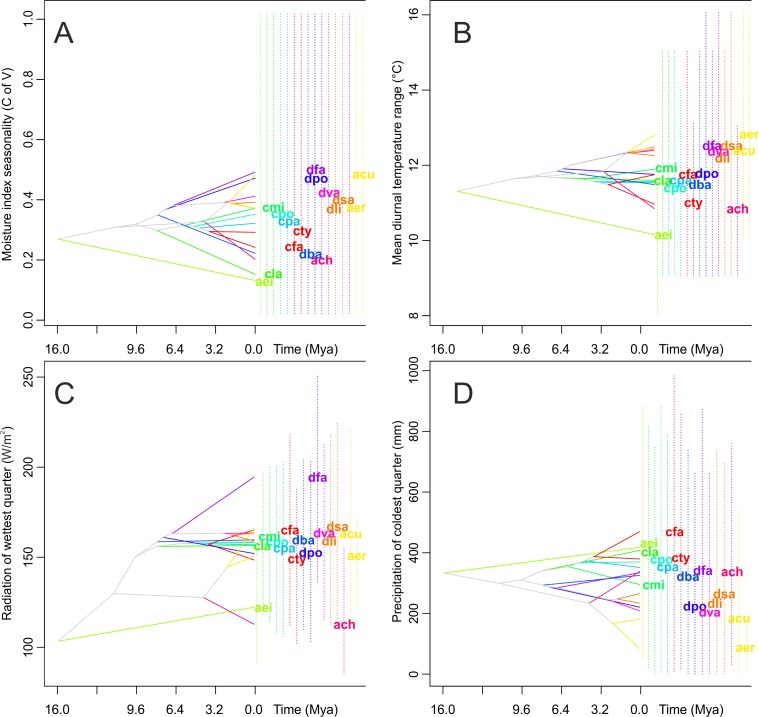
Examples of evolution of climatic tolerances in Angraecinae visualized on the consensus tree from Bayesian inference. (A) Moisture index seasonality (bioclim 31); (B) Mean diurnal temperature range (bioclim 2); (C) Radiation of wettest quarter (bioclim 24); and (D) Precipitation of coldest quarter (bioclim 19). The name of each studied taxon was abbreviated to the first letter of generic name and two first letters of specific epithet (e.g., *Campylocentrum fasciola* code is (“cfa”)). Interior nodes represent the mean of climatic tolerances inferred for the most recent common ancestor of the extant taxa defined by that node. The 80% central density of climatic tolerance for each extant taxon is indicated by a vertical dashed line, and the mean is indicated by the taxon label, to the right of each graph. For visualization the four most important uncorrelated variables were chosen as indicated by Maxent analysis on a full set of 35 bioclimatic variables.

There are no significant differences in the evolutionary history of the taxa studied in their climatic suitabilities and tolerance of species overlap (Fig. 7). Species of both the Neotropical genera included in this study appeared to have slightly wider climatic suitabilities than their closest African relatives. The climatic suitability of *Angraecum eichlerianum* measured in terms of the climatic factors analyzed has changed very little over time. The niches of the other species of *Angraecum* studied (*A. erectum*, *A. chevalieri* and *A. cultriforme*) diverged and stabilized much later. The climatic suitability of the Neotropical species evolved in various directions and the climatic niche was apparently not highly conserved within particular genera. Climatic suitability of the African *Angraecum cultriforme* and *A. erectum* changed and became more similar to that of Neotropical Angraecinae.

**Figure 8:**
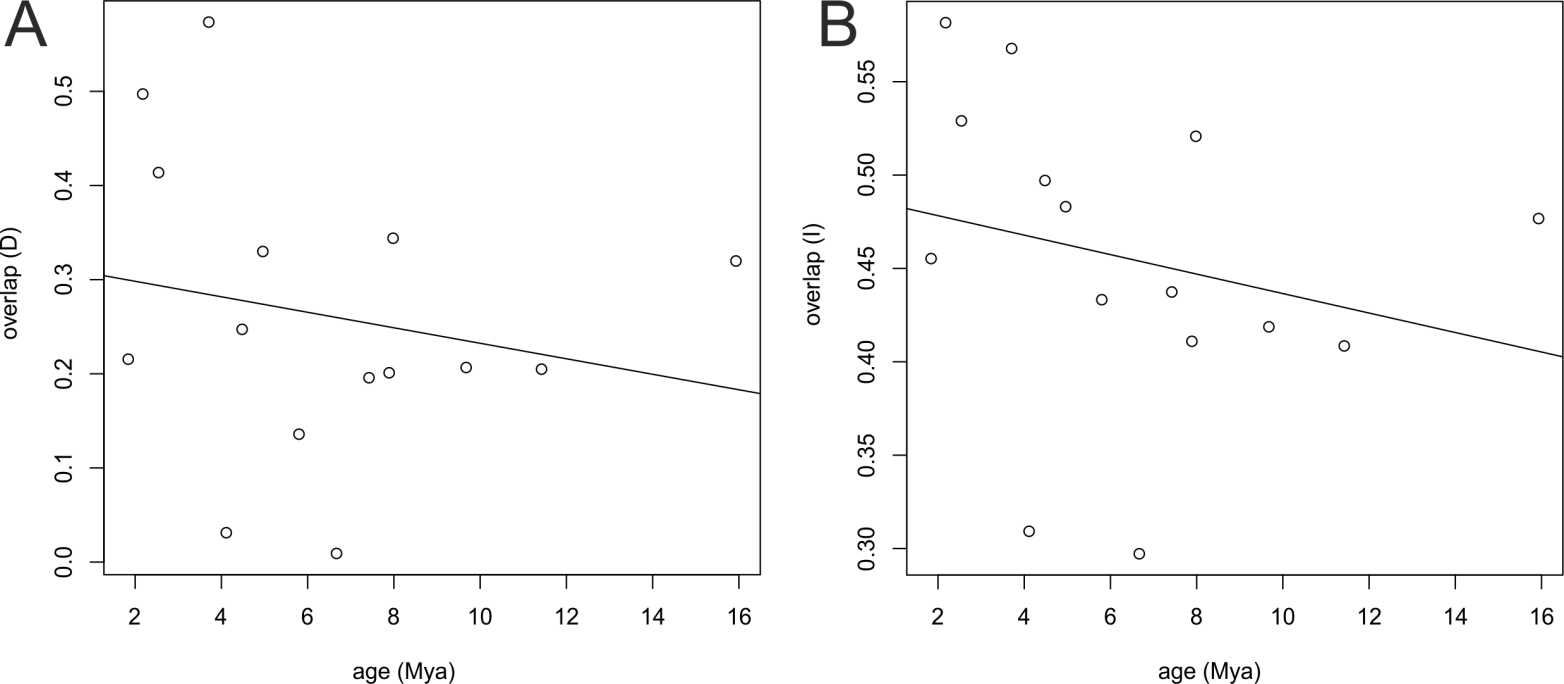
Result of age range correlation (ARC) analysis which presents phylogenetic signal of niche similarity for both genera using D statistic (A) or I statistic (B) in function of time (Mya). Each dot represents a node of the phylogenetic tree (Fig. 1) and the dots on the right side represent the ancestral nodes. Line is the fitted regression.

Similar results were obtained from the age range correlation using both D and I for which the slope is negative and intercept is higher than 0.5, which could indicate sympatric speciation (Fitzpatrick & Turelli, 2006; Warren, Glor & Turelli, 2008). These results imply that recently diverged nodes are more similar than more ancestral nodes and recent species occupy similar niches (Fig. 8). ARC was not significant (Table 2), which indicates that climatic niche differentiation did not play a role in the diversification of Angraecinae. The evolution of Angraecinae corresponds to Brownian motion. The species in this group do not exhibit ecological specialization within their climatic suitabilities.

**Table 2:** Results of the age-range correlation (ARC) analyses using randomization tests under Monte Carlo resampling.

	Intercept	f (greater)^1^	Slope	f (greater)^1^
D	0.6747	0.055 (*P* = 0.11)	−0.2719	0.89 (*P* = 0.23)
I	0.8528	0.13 (*P* = 0.25)	−0.1727	0.79 (*P* = 0.42)

## Discussion

### Phylogenetic niche conservatism

Traditionally, natural selection and adaptation result in lineages inhabiting different environments. However, the similarity between the niches occupied by Neotropical angrecoid orchids and their African relatives indicated by this research suggests that they have retained the general climatic suitability of their common progenitor. The recorded shift in climatic suitability of two ancestral African angrecoid orchids could have occurred relatively recently, after the stabilization of niche preferences within New World species of this subtribe. Another possible explanation is that their climatic suitabilities were derived from that of the ancestor of these taxa.

Wiens (2004) suggests that limited adaptation to environmental conditions is a crucial factor in promoting the divergence in the initial origin of lineages and less important in the subsequent divergence of these lineages. The results of this study reveal that climatic niche diversification did not significantly influence the speciation of the Neotropical angrecoid orchids. This is the third study showing that PNC in Orchidaceae is very high (Kolanowska & Szlachetko, 2014; Kolanowska et al., 2016) and that their diversity cannot be explained by niche differentiation. Our results confirm Wiens’s assumption about the low effect of niche modification in speciation.

### Evolutionary implications

The divergence of Neotropical angrecoid orchids from their African relatives took place ca. 11.5–16 Mya (Micheneau et al., 2010), in the Miocene, but the diversification within the American representatives of Angraecinae began about 6–7 Mya. The late Miocene marked the start of a paroxysm resulting in the uplift of the northeastern Andes (Hoorn et al., 1995) and a significant increase in sediment rates between ca. 7.9 and 6 Mya (Uba, Strecker & Schmitt, 2007). The Andean sediments reached the Atlantic coast via the Amazon drainage system, and the Amazon River became fully established at about 7 Mya (Hoorn et al., 2010). The rise of the Andes influenced climatic conditions in South America and the range began to constitute a solid migration barrier that limited the dispersal of *Campylocentrum*. Uplift of the Lesser Antilles arc and the associated platform initiated during Early-Middle Miocene formed a barrier to sedimentation between the two basins by the late Miocene. After the Miocene, most deposition in the Greater Antilles was confined to present coastal and offshore areas (Khudoley & Meyerhoff, 1971). This heterogeneity in topography and environmental conditions probably led to rapid divergence between populations, as it reduced the dispersal and gene flow between adjacent populations. Based on the geological events that occurred at the time of diversification of Neotropical Angraecinae and the result of the ENM analysis we assume that adaptation to various niches was not the main factor determining speciation within the taxa studied.

The climatic-based potential ranges of the Neotropical species studied are considerably larger than the observed distributions of these orchids, indicating that abiotic conditions are not the main factor limiting the occurrence of *Campylocentrum* and *Dendrophylax*. Whilst the species of these two genera are characterized by similar climatic suitabilities and they share the same evolutionary and geological history, their known geographical ranges differ significantly. Populations of *Campylocentrum* occur on both sides of the Andes as well as in Mesoamerica and the West Indies. The distribution of *Dendrophylax* is restricted to Mesoamerica and the West Indies. Since there is no report of a firm relationship between any of the American angrecoid orchids and a specific phorophyte, we believe that only two factors could promote the diversification of these two genera: pollinator specificity and/or mycorrhizal specialization. Unfortunately, little is known about both of these two aspects of angrecoid orchid biology. Preliminary studies reveal that some species of *Campylocentrum* form mycorrhizal associations with Ceratobasidiaceae (Otero, Ackerman & Bayman, 2002) and it is possible that the same symbiotic association occurs in *Dendophylax lindenii* (Chomicki, Bidel & Jay-Allemand, 2014). Undoubtedly, leafless species rely on endomycorrhizal associations, but the diversity of their mycobionts remains unknown.

Pollinator specificity is suggested as a promoter of evolution and speciation in angraecoid orchids by Dressler (1981). Both *Capylocentrum* and *Dendrophylax* are entomophilous, although flowers of *Dendrophylax* are reported to be pollinated by sphignid moths and the pollen of *Campylocentrum* is also transferred by halictid and meliponini bees (Singer & Cocucci, 1999; Singer, 2003). Whilst species of *Campylocentrum* grow sympatrically (Pupulin, 1998; Pupulin, 2001; Bogarín & Pupulin, 2010), there are no records of hybrids between any the Neotropical angrecoid orchids. The lack of any significant differences in climatic suitability is also recorded in the African *Angraecum*, a close relative of Neotropical Angraecinae. The African angrecoids are highly specialized in terms of moth pollination. Most of *Angraecum* species produce long-spurred, white, nectariferous flowers. These floral characters are consistent with hawkmoth pollination (Darwin, 1862; Grant, 1985; Nilsson et al., 1985; Haber & Frankie, 1989; Micheneau et al., 2008). However, long-spurred species, especially those in Madagascar, are recognized as ancestral in the angraecoid orchid group (Nilsson et al., 1985). The studies on the pollination of species of *Angraecum* have mainly been on the Malgasy (Nilsson et al., 1985; Arditti et al., 2012) and Mascarene Islands (Micheneau, Fournel & Pailler, 2006; Micheneau et al., 2008). Moreover, the research of Micheneau et al. (2008) indicates that species of section *Hadrangis,* which have atypical short-spurred, scentless flowers are pollinated by birds and crickets. Unfortunately, the data on pollination are still very scanty and the relationship with pollinators of *Angraecum* species on Mainland Africa has not been studied. Most likely differences in pollinator composition provides a strong barrier to gene flow.

In conclusion, we found that niche conservatism in Angrecinae is very strong and that the African and Neotropical species in this group have similar climatic suitabilities. Moreover, climatic niche differentiation does not appear to be an important factor in the speciation of *Dendrophylax* and *Campylocentrum*. We suggest that pollinator specificity or restricted mycorrhizal associations played a crucial role in the development of the diversity of species in the taxa studied.

##  Supplemental Information

10.7717/peerj.3328/supp-1 Table S1 List of localities used in the ENM analysis

10.7717/peerj.3328/supp-2 Table S2 PCA loadings obtained after creating PCA derived maps. The first six components were used in the final analysis

10.7717/peerj.3328/supp-3 Table S3 Genbank numbers of the sequences used in the phylogenetic analysis

## Additional Information and Declarations

### Data Availability

The following information was supplied regarding data availability: The raw data has been supplied as a  Supplementary File.
