# Supplementary material for: Phylogenetic climatic niche conservatism and evolution of climatic suitability in Neotropical Angraecinae (Vandeae, Orchidaceae) and their closest African relatives
Source: PeerJ. 2017 May 16;5:e3328. doi: 10.7717/peerj.3328 (PMC5436590; doi:10.7717/peerj.3328)
Supplement: Table S1 [file peerj-05-3328-s001.doc]

**Table S1. List of localities used in the ENM analysis.** Acronyms of herbaria names follows Thiers 2015.

| **Species** | **Country** | **Collector** | **Source (reference/herbarium)** | **Coordinates** | **DATUM** |
| --- | --- | --- | --- | --- | --- |
| *Campylocentrum fasciola* | Costa Rica | Bogarín 2220 | Bogarin & Pupulin 2010 | -84.5232222222222;10.6216944444444 | WGS1984 |
| *Campylocentrum fasciola* | Costa Rica | Bogarín 4245 1119 | Bogarin & Pupulin 2010 | -83.6677777777778;9.90083333333333 | WGS1984 |
| *Campylocentrum fasciola* | Costa Rica | Ossenbach 336 | Bogarin & Pupulin 2010 | -84.0167416666667;10.4735222222222 | WGS1984 |
| *Campylocentrum fasciola* | Costa Rica | Bogarín 4245 | Bogarin & Pupulin 2010 | -83.6130555555555;10.0440833333333 | WGS1984 |
| *Campylocentrum fasciola* | Brazil | Milliken & al. 154 | K | -61.5166666666667;3.35 | WGS1984 |
| *Campylocentrum fasciola* | Suriname | Kappler 1660 | K | -55.2260444444444;5.48175277777778 | WGS1984 |
| *Campylocentrum lansbergii* | Colombia | Idrobo, Cleef, Rangel & Salamanca 9916 | COL | -75.6212611111111;4.86516666666667 | WGS1984 |
| *Campylocentrum lansbergii* | Colombia | Gonzalez YGO2018 | COL | -73.0937444444444;7.05145277777778 | WGS1984 |
| *Campylocentrum lansbergii* | Venezuela | Steyermark, Liesner & Delascio 114478 | MO | -61.7638888888889;9.68055555555556 | WGS1984 |
| *Campylocentrum lansbergii* | Venezuela | Liesner 19637 | MO | -61.5833333333333;4.5 | WGS1984 |
| *Campylocentrum lansbergii* | Venezuela | Steyermark, Liesner & Delascio 114979 | MO | -60.95;9.25472222222222 | WGS1984 |
| *Campylocentrum micranthum* | Venezuela | Steyermark 88558 | W | -61.3333333333333;7.6667 | WGS1984 |
| *Campylocentrum micranthum* | Guyana | Fenshawe F2380 | K | 0;0 | WGS1984 |
| *Campylocentrum micranthum* | Colombia | Orozco, Rivera, Torrez, Lozano & Pinto 654 | COL | -74.9966444444444;6.03646388888889 | WGS1984 |
| *Campylocentrum micranthum* | Colombia | Uribe Uribe 3116 | COL | -76.6053194444444;5.71896666666667 | WGS1984 |
| *Campylocentrum micranthum* | Colombia | Chaparro de Barrera & Barrera Torres 312 | COL | -74.4334555555556;4.96535277777778 | WGS1984 |
| *Campylocentrum micranthum* | Costa Rica | Bogarín 696 | Bogarin & Pupulin 2010 | -83.6677777777778;9.90083333333333 | WGS1984 |
| *Campylocentrum micranthum* | Costa Rica | Bogarín 1272 | Bogarin & Pupulin 2010 | -84.5681111111111;9.72827777777778 | WGS1984 |
| *Campylocentrum micranthum* | Bolivia | Vargas, Balcazar & Tagua 2538 | MO | -63.9733333333333;-4.725 | WGS1984 |
| *Campylocentrum micranthum* | Brazil | Assunção & Silva 851 | K | -59.9666666666667;-26.9033333333333 | WGS1984 |
| *Campylocentrum micranthum* | Peru | Percy Núñez 6870 | MO | -71.5916666666667;-13.3155694444444 | WGS1984 |
| *Campylocentrum pachyrrhizum* | French Guiana | - | Chiron & Bellone 2005 | -53.2;3.61661666666667 | WGS1984 |
| *Campylocentrum pachyrrhizum* | Jamaica | J.P. 2326 | Fawcett & Rendle 1982 | -76.8252888888889;18.2020694444444 | WGS1984 |
| *Campylocentrum pachyrrhizum* | Tobago | Broadway 2477 | Cogniaux 1909 | -60.7159916666667;11.1971805555556 | WGS1984 |
| *Campylocentrum pachyrrhizum* | Jamaica | Morris 2326 | Cogniaux 1909 | -76.4259388888889;17.88095 | WGS1984 |
| *Campylocentrum poeppigii* | Jamaica | Moore s.n. | Fawcett & Rendle 1979 | -76.5647138888889;18.1984611111111 | WGS1984 |
| *Campylocentrum poeppigii* | Costa Rica | Horich s.n. | Bogarin & Pupulin 2011 | -84.3539194444444;10.6593083333333 | WGS1984 |
| *Campylocentrum poeppigii* | Costa Rica | Bogarín 2218 | Bogarin & Pupulin 2011 | -84.5215555555556;10.6216944444444 | WGS1984 |
| *Campylocentrum poeppigii* | Guatemala | Contreras 1960 | LL | -89.6797222222222;17.1372222222222 | WGS1984 |
| *Campylocentrum poeppigii* | Brazil | Ratter & al.. 6222 | K | -61.5166666666667;3.35 | WGS1984 |
| *Campylocentrum sullivanii (C. fasciola)* | Jamaica | Harris 7523 | Fawcett & Rendle 1980 | -76.4259388888889;17.8810583333333 | WGS1984 |
| *Campylocentrum sullivanii (C. fasciola)* | Jamaica | Sullivan s.n. | Fawcett & Rendle 1981 | -77.4819694444444;18.1666277777778 | WGS1984 |
| *Campylocentrum sullivanii (C. fasciola)* | Jamaica | Wilson s.n. | Fawcett & Rendle 1982 | -78.3472361111111;18.2683027777778 | WGS1984 |
| *Campylocentrum tyrridion* | Jamaica | Moulton-Barrett, | K | -77.3663833333333;18.3938833333333 | WGS1984 |
| *Campylocentrum tyrridion* | Mexico | Carnevali & Chi 5127 | Romero & Carnevali 2005 | -88.5472222222222;19.0994444444444 | WGS1984 |
| *Campylocentrum tyrridion* | Ecuador | Holm-Nielsen & al.. 2719 | Romero & Carnevali 2005 | -1.78333333333333;-79.2833333333333 | WGS1984 |
| *Campylocentrum tyrridion* | Ecuador | Dodson & al.. 14129 | Romero & Carnevali 2005 | -79.363625;-0.53165 | WGS1984 |
| *Campylocentrum tyrridion* | Panama | Duke 4865 | MO | -77.7333333333333;8.1167 | WGS1984 |
| *Dendrophylax barrettiae* | Jamaica | Barrett s.n. | Fawcett & Rendle 1982 | -77.3663833333333;18.3938833333333 | WGS1984 |
| *Dendrophylax barrettiae* | Jamaica | Harris s.n. | Fawcett & Rendle 1982 | -77.0961333333333;18.2231666666667 | WGS1984 |
| *Dendrophylax barrettiae* | Dominican Republic | Alain & Marcano 14744 | AMES | -70.6770833333333;18.8428861111111 | WGS1984 |
| *Dendrophylax fawcetti* | Cayman Islands | Dressler 2904 | GH | -81.3675416666667;19.2944083333333 | WGS1984 |
| *Dendrophylax fawcetti* | Cayman Islands | Kings GC19 | MO | -81.4155972222222;19.3768666666667 | WGS1984 |
| *Dendrophylax fawcetti* | Dominican Republic | Alain & Marcano 15033 | AMES | -69.6183166666667;18.8114166666667 | WGS1984 |
| *Dendrophylax lindenii* | USA | Lassiter 15 | FLAS | -81.4098611111111;25.995325 | WGS1984 |
| *Dendrophylax lindenii* | USA | Cooley 792 | FLAS | -81.0287138888889;25.8636861111111 | WGS1984 |
| *Dendrophylax lindenii* | Cuba | Hioram 7603 | US | -75.2419027777778;20.1918888888889 | WGS1984 |
| *Dendrophylax lindenii* | Cuba | Wright 3303 | ? | -76.4441694444445;20.9878444444444 | WGS1984 |
| *Dendrophylax lindenii* | Cuba | Roig & al.. 9542 | AMES | -76.8366694444444;19.9896638888889 | WGS1984 |
| *Dendrophylax porrectus* | Mexico | Carnevali & al.. 4468 | AMO | 19; -90.30 | WGS1984 |
| *Dendrophylax porrectus* | Mexico | Duran & Olmsted 1020 | MEXU | -88.3;19.4 | WGS1984 |
| *Dendrophylax porrectus* | Mexico | Gaumer & et al. 23359 | F | -89.19;20.68 | WGS1984 |
| *Dendrophylax porrectus* | Mexico | Matuda 18674 | AMES | -92.52;15.44 | WGS1984 |
| *Dendrophylax porrectus* | USA | Brewer s.n. | FLAS | -81.3397722222222;26.0526611111111 | WGS1984 |
| *Dendrophylax porrectus* | Dominican Republic | Whitten & al.. 1950 | FLAS | -71.086028;18.759139 | WGS1984 |
| *Dendrophylax porrectus* | El Salvador | Hamer 442 | AMES | -89.14;13.42 | WGS1984 |
| *Dendrophylax porrectus* | Puerto Rico | Ackerman & Roubik 2715 | NY | -66.8618;18.0297611111111 | WGS1984 |
| *Dendrophylax sallei* | Dominican Republic | Howard & Howard 8675 | AMES | -71.2904;18.8956305555556 | WGS1984 |
| *Dendrophylax sallei* | Dominican Republic | Alain 13319 | GH | -70.6871083333333;19.7816611111111 | WGS1984 |
| *Dendrophylax sallei* | Dominican Republic | Mejia & al.. 973 | GH | -70.3836444444445;18.2853055555556 | WGS1984 |
| *Dendrophylax sallei* | Dominican Republic | Türckheim 3281 | GH | -70.5311583333333;19.22605 | WGS1984 |
| *Dendrophylax sallei* | Dominican Republic | Alain 14699 | AMES | -71.2410972222222;18.7905666666667 | WGS1984 |
| *Dendrophylax varius* | Dominikan Republic | Liogier 15274 | NY | -70.96455;19.6442333333333 | WGS1984 |
| *Dendrophylax varius* | Cuba | Shafer 2831 | AMES | -77.9038638888889;21.388625 | WGS1984 |
| *Dendrophylax varius* | Dominikan Republic | Ekman H13469 | GH | -70.7400222222222;18.4599527777778 | WGS1984 |
| *Dendrophylax varius* | Haiti | Ekman 894 | GH | -72.5003972222222;18.9178222222222 | WGS1984 |
| *Dendrophylax varius* | Cuba | Wright 3300 | GH | 0;0 | WGS1984 |
| *Dendrophylax varius* | Cuba | Smith & al.. 3321 | GH | 0;0 | WGS1984 |
| *Dendrophylax varius* | Haiti | Christ 2053 | Urban 1918 | -72.2437805555556;19.5269638888889 | WGS1984 |
| *Angraecum chevalieri* | Ivory Coast | Chevalier 21690 | P | -5.73333333333333;8.28333333333333 | WGS1984 |
| *Angraecum chevalieri* | Cameroon | Brunt 1212 | K | 10.2480361111111;6.07925 | WGS1984 |
| *Angraecum chevalieri* | Cameroon | Letouzey 2188 | K | 12.1217861111111;5.43988888888889 | WGS1984 |
| *Angraecum chevalieri* | Cameroon | Letouzey 11583 | P | 11.5;3.51666666666667 | WGS1984 |
| *Angraecum chevalieri* | Cameroon | Sanford 5215 | K | 12.1343138888889;3.76900833333333 | WGS1984 |
| *Angraecum chevalieri* | Cameroon | Sanford 5234 | P | 13.1666666666667;3.98333333333333 | WGS1984 |
| *Angraecum chevalieri* | Cameroon | Sanford 6142 | P | 13.5666666666667;4.38333333333333 | WGS1984 |
| *Angraecum chevalieri* | Cameroon | Stévart & Pial 509 | P | 14.01944;3.825 | WGS1984 |
| *Angraecum chevalieri* | Central African Republic | Tisserant 2071 | K | 17.9153305555556;3.89293333333333 | WGS1984 |
| *Angraecum chevalieri* | Equatorial Guinea (Bioko) | Sanford 5738 | K | 10.8400638888889;2.12198611111111 | WGS1984 |
| *Angraecum chevalieri* | Liberia | de Konig 528 | WAG | -8.1833333333;6.1666666667 | WGS1984 |
| *Angraecum chevalieri* | Liberia | Harley 1637 | L | -8.9833333333;7.2333333333 | WGS1984 |
| *Angraecum chevalieri* | Ghana | Johnson | LEGON-GC | -0.11;5.51 | WGS1984 |
| *Angraecum chevalieri* | Togo | Brunel 7311 | TOGO | 0.6;7.5833333 | WGS1984 |
| *Angraecum chevalieri* | Gabon | Le Testu 9271 | BM | 11.6166666667;1.8333333333 | WGS1984 |
| *Angraecum chevalieri* | Gabon | Reitsma & Louis 1799 | WAG | 11.0166666667;0.9166666667 | WGS1984 |
| *Angraecum chevalieri* | Gabon | Stévart 1828 | L | 11.6280833333;1.82615 | WGS1984 |
| *Angraecum chevalieri* | Gabon | Stévart, & Biteau 7 | L | 11.5833333333;1.6166666667 | WGS1984 |
| *Angraecum chevalieri* | Gabon | Deman, V 72 | L | 11.62525;1.8274166667 | WGS1984 |
| *Angraecum erectum* | Zambia | Richards 11785 | K | 32.2666666666667;-8.88333333333333 | WGS1984 |
| *Angraecum erectum* | Zambia | Richards 6973 | K | ; | WGS1984 |
| *Angraecum erectum* | Tanzania | Moreau 308a | K | 35.8;-3.25 | WGS1984 |
| *Angraecum erectum* | Tanzania | Moreau 632 | K | 35.6166666666667;-2.05 | WGS1984 |
| *Angraecum erectum* | Tanzania | Moreau 308 | K | 34.9333333333333;-4.23333333333333 | WGS1984 |
| *Angraecum erectum* | Tanzania | Pocs, & Chuwa 89036 | K | 35.3027222222222;-3.92905833333333 | WGS1984 |
| *Angraecum erectum* | Tanzania | Carmichael 1302 | K | 36.2166666666667;-2.9 | WGS1984 |
| *Angraecum erectum* | Kenya | Copley 20 | K | 36.5391777777778;0.267136111111111 | WGS1984 |
| *Angraecum erectum* | Kenya | Tweedie 386 | K | 35.5192638888889;0.59185 | WGS1984 |
| *Angraecum erectum* | Kenya | Schelpe  s.n. | K | 37.4476694444444;-0.158761111111111 | WGS1984 |
| *Angraecum erectum* | Kenya | Greenway 10224 | K | 35.8666638888889;-1.08332222222222 | WGS1984 |
| *Angraecum erectum* | Kenya | Kårehed & Odhult | UPS | 35.8463;-1.8448 | WGS1984 |
| *Angraecum erectum* | Kenya | Bally 5632 | K | 36.9346611111111;2.75041666666667 | WGS1984 |
| *Angraecum erectum* | Kenya | Cunningham-van Someren 51 | K | 36.8304111111111;-1.17481111111111 | WGS1984 |
| *Angraecum erectum* | Kenya | Gilbert, Gachathi & Gatheri 5121 | K | 36.7018305555556;1.09314166666667 | WGS1984 |
| *Angraecum erectum* | Kenya | Tweedie 379 | K | 35.2833333333333;0.516666666666667 | WGS1984 |
| *Angraecum erectum* | Kenya | Someren 8455 | K | 36.9569444444444;-1.14888888888889 | WGS1984 |
| *Angraecum erectum* | Kenya | Polhill 216 | K | 36.8396694444444;-1.23354444444444 | WGS1984 |
| *Angraecum erectum* | Kenya | Moreau 579 | K | 37.0666666666667;0.0166666666666667 | WGS1984 |
| *Angraecum erectum* | Kenya | Glover 3042 | K | ; | WGS1984 |
| *Angraecum erectum* | Kenya | Bally 7532 | K | 36.7818666666667;-1.295975 | WGS1984 |
| *Angraecum erectum* | Kenya | Cunningham-van Someren 56 | K | 36.7387194444444;-1.3633 | WGS1984 |
| *Angraecum erectum* | Kenya | Khayota 125 | K | 36.6417777777778;-1.41525555555556 | WGS1984 |
| *Angraecum cultriforme* | Kenya | Robertson & Luke 5744 | MO | 39.2;-4.52 | WGS1984 |
| *Angraecum cultriforme* | South Africa | Harrison s.n. | PRE | 28.125;-32.375 | WGS1984 |
| *Angraecum cultriforme* | South Africa | Buthelezi | SANBI | 27.625;-32.625 | WGS1984 |
| *Angraecum cultriforme* | South Africa | Jordaan | SANBI | 27.6250001;-32.6250001 | WGS1984 |
| *Angraecum cultriforme* | South Africa | Ward | SANBI | 27.125;-32.625 | WGS1984 |
| *Angraecum cultriforme* | Tanzania | FRONTIER-TANZANIA 2520 | MO | 38.1477277777778;-5.45285277777778 | WGS1984 |
| *Angraecum cultriforme* | Tanzania | Milne-Redhead & Taylor 7318 | K | 38.6444638888889;-5.57434722222222 | WGS1984 |
| *Angraecum cultriforme* | Malawi | la Croix & Spurrier 779 | K | 35.4879416666667;-16.0836583333333 | WGS1984 |
| *Angraecum cultriforme* | Mozambique | Gomes e Sousa 4299 | K | ; | WGS1984 |
| *Angraecum cultriforme* | Zambia | Brenan 5 | K | 28.2438916666667;-15.3585361111111 | WGS1984 |
| *Angraecum cultriforme* | Zimbabwe | Ball 945 | SRGH | 32.8532;-18.4967777777778 | WGS1984 |
| *Angraecum eichlerianum* | Cameroon | Bates 1642 | BM | 12.3511694444444;3.01152222222222 | WGS1984 |
| *Angraecum eichlerianum* | Cameroon | Etuge & Thomas 194 | K | 9.55;4.71666666666667 | WGS1984 |
| *Angraecum eichlerianum* | Cameroon | Jacques-Felix 2905 | P | 10.257525;5.09973055555556 | WGS1984 |
| *Angraecum eichlerianum* | Cameroon | Leeuwenberg 8208 | WAG | 9.9;4.83333333333333 | WGS1984 |
| *Angraecum eichlerianum* | Cameroon | Letouzey 15120 | K | 8.88004444444445;5.14617777777778 | WGS1984 |
| *Angraecum eichlerianum* | Cameroon | Merle 8 | P | 9.62856666666667;4.55441388888889 | WGS1984 |
| *Angraecum eichlerianum* | Cameroon | Nemba & Thomas 152 | K | 9.48333333333333;4.75 | WGS1984 |
| *Angraecum eichlerianum* | Cameroon | Thomson & Rawlins 1428 | K | 9.03333333333333;4.06666666666667 | WGS1984 |
| *Angraecum eichlerianum* | Cameroon | Thomson & Rawlins 1588 | K | 9;4.18333333333333 | WGS1984 |
| *Angraecum eichlerianum* | Cameroon | Thorold 34 | K | 8.97861111111111;4.10083333333333 | WGS1984 |
| *Angraecum eichlerianum* | Cameroon | Villiers 936 | P | 10.275;3.175 | WGS1984 |
| *Angraecum eichlerianum* | Gabon | Breteler & De Wilde 546 | WAG | 13.1833333333;1.0666666667 | WGS1984 |
| *Angraecum eichlerianum* | Gabon | de Wilde, Arends & de Bruijn 9185 | K | 10.6666666666667;-3.35 | WGS1984 |
| *Angraecum eichlerianum* | Gabon | Le Testu 5756 | BM | 11.9166666667;-1.9166666667 | WGS1984 |
| *Angraecum eichlerianum* | Gabon | Halle 3068, 3512, 3615 & 3774 | P | 13.1333333333;1.0833333333 | WGS1984 |
| *Angraecum eichlerianum* | Gabon | Reitsma 3015 | MA | 11.0033333333333;0.75 | WGS1984 |
| *Angraecum eichlerianum* | Gabon | Lejoly, & Ngok Banak 1342 | WAG | 11.6298;1.6469666667 | WGS1984 |
| *Angraecum eichlerianum* | Gabon | Strijk 310 | WAG | 11.7166666667;1.0666666667 | WGS1984 |
| *Angraecum eichlerianum* | Gabon | de Wilde 9185 | WAG | 10.6666666667;-3.35 | WGS1984 |
| *Angraecum eichlerianum* | Gabon | Louis 2219 | WAG | 10.6666666667;-3.4166666667 | WGS1984 |
| *Angraecum eichlerianum* | Gabon | Moungazi & Ngok Banak 1104 | WAG | 10.8992166667;0.9793833333 | WGS1984 |
| *Angraecum eichlerianum* | Gabon | Stévart 876 | WAG | 10.4070166667;0.63155 | WGS1984 |
| *Angraecum eichlerianum* | Angola | Gossweiler 13898 | K | 20.81955;-7.37416944444444 | WGS1984 |
| *Angraecum eichlerianum* | Congo | Sita 3220 | WAG | 13.8333333333;-2.85 | WGS1984 |
| *Angraecum eichlerianum* | Congo | Trochain 8137 | P | 14.9747222222222;4.58416666666667 | WGS1984 |
| *Angraecum eichlerianum* | DRC | Louis 5616 | BR | 24.4414111111111;0.767355555555556 | WGS1984 |
| *Angraecum eichlerianum* | DRC | Louis 5673 | BR | 24.4414111111111;0.767355555555556 | WGS1984 |
| *Angraecum eichlerianum* | DRC | Louis 8491 | BR | 24.4414111111111;0.767355555555556 | WGS1984 |
| *Angraecum eichlerianum* | DRC | Louis 13674 | BR | 20.5333055555556;-1.43353888888889 | WGS1984 |
| *Angraecum eichlerianum* | DRC | Nsimundele 879 | BR | 12.77;-5.82 | WGS1984 |
| *Angraecum eichlerianum* | DRC | Evrard 4961 | BR | 20.9;0.5 | WGS1984 |
| *Angraecum eichlerianum* | DRC | Louis 2597 | BR | 24.45;0.77 | WGS1984 |
| *Angraecum eichlerianum* | DRC | Louis 16047 | BR | 24.5;0.93 | WGS1984 |
| *Angraecum eichlerianum* | DRC | Lisowski 16809 | BR | 25.15;0.93 | WGS1984 |
| *Angraecum eichlerianum* | DRC | Hauman 661 | BR | 22.08;1.4 | WGS1984 |
| *Angraecum eichlerianum* | DRC | Solheid 3 | BR | 24.55;1.27 | WGS1984 |
| *Angraecum eichlerianum* | DRC | Laurent 1067 | BR | 24.55;1.27 | WGS1984 |
| *Angraecum eichlerianum* | DRC | Louis 7862 | BR | 24.52;1.05 | WGS1984 |
| *Angraecum eichlerianum* | DRC | Le Jeune 20 | BR | 22.98;2.22 | WGS1984 |
| *Angraecum eichlerianum* | DRC | Pynaert 94 | BR | 22.53;2.18 | WGS1984 |
| *Angraecum eichlerianum* | DRC | Gérard 3907 | BR | 25.72;3.47 | WGS1984 |
| *Angraecum eichlerianum* | DRC | Gérard 4563 | BR | 25.72;3.47 | WGS1984 |
| *Angraecum eichlerianum* | DRC | Gérard 4931 | BR | 25.72;3.47 | WGS1984 |
| *Angraecum eichlerianum* | DRC | Gérard 5236 | BR | 25.72;3.47 | WGS1984 |
| *Angraecum eichlerianum* | DRC | Seret 655B | BR | 26.88;-3.15 | WGS1984 |
| *Angraecum eichlerianum* | DRC | Villiers 1515 | HNC | 9.82096111111111;4.89558333333333 | WGS1984 |
| *var.curvicalcaratum* | Cameroon | Annet 1475 | P | 10.07275;3.48962222222222 | WGS1984 |
| *var.curvicalcaratum* | Cameroon | Stevart, Chamba & Pial 105 | BRLU | 12.7067166666667;3.31705 | WGS1984 |
| *var.curvicalcaratum* | Cameroon | Stevart, Chamba & Pial 106 | BRLU | 12.79075;3.32205 | WGS1984 |
| *var.curvicalcaratum* | Cameroon | Stevart, Chamba & Pial 123 | BRLU | 12.79075;3.32205 | WGS1984 |
| *var.curvicalcaratum* | Cameroon | Stevart, Chamba & Pial 129 | BRLU | 12.79075;3.32205 | WGS1984 |
| *var.curvicalcaratum* | Cameroon | Stevart, Chamba & Pial 126 | BRLU | 12.7067166666667;3.31705 | WGS1984 |
| *var.curvicalcaratum* | Cameroon | Stevart, Chamba & Pial 159 | BRLU | 12.7067166666667;3.31705 | WGS1984 |
| *var.curvicalcaratum* | Cameroon | Stevart, Chamba & Pial 166 | BRLU | 12.7067166666667;3.31705 | WGS1984 |
| *var.curvicalcaratum* | Cameroon | Stevart, Chamba & Pial 167 | BRLU | 12.7067166666667;3.31705 | WGS1984 |
| *var.curvicalcaratum* | Cameroon | Stevart, Chamba & Pial 387 | BRLU | 12.7913;3.28608333333333 | WGS1984 |
| *var.curvicalcaratum* | Cameroon | Stevart, Chamba & Pial 395 | BRLU | 12.7914166666667;3.30115 | WGS1984 |
| *var.curvicalcaratum* | Cameroon | Stevart, Chamba & Pial 404 | BRLU | 12.7911166666667;3.32178333333333 | WGS1984 |
| *var.curvicalcaratum* | Cameroon | Stevart, Chamba & Pial 420 | BRLU | 12.9555666666667;2.86821666666667 | WGS1984 |
| *var.curvicalcaratum* | Cameroon | Stevart, Chamba & Pial 425 | BRLU | 12.9870333333333;2.99362222222222 | WGS1984 |
| *var.curvicalcaratum* | Cameroon | Stevart, Chamba & Pial 456 | BRLU | 12.9445;2.86563333333333 | WGS1984 |
| *var.curvicalcaratum* | Cameroon | Stevart, Chamba & Pial 481 | BRLU | 12.79075;3.32205 | WGS1984 |
| *var.curvicalcaratum* | Cameroon | Stevart, Chamba & Pial 484 | BRLU | 12.79075;3.32205 | WGS1984 |
| *var.curvicalcaratum* | Cameroon | Stevart, Chamba & Pial 544 | BRLU | 12.71025;3.31576666666667 | WGS1984 |
| *var.curvicalcaratum* | Cameroon | Stevart, Droissart & Simo 2139 | BRLU | 9.74986666666667;5.59536666666667 | WGS1984 |
| *var.curvicalcaratum* | Cameroon | Droissart 21 | BRLU | 11.36715;3.92066666666667 | WGS1984 |
| *var.curvicalcaratum* | Cameroon | Droissart 67 | BRLU | 11.3701666666667;3.92573333333333 | WGS1984 |
| *var.curvicalcaratum* | Cameroon | Stevart, Droissart & Simo 394 | BRLU | 11.36715;3.92066666666667 | WGS1984 |
| *var.curvicalcaratum* | Cameroon | Droissart, Stevart & Simo 396 | BRLU | 12.79075;3.32205 | WGS1984 |
| *var.curvicalcaratum* | Cameroon | Droissart, Stevart & Simo 400 | BRLU | 10.4090666666667;3.18511666666667 | WGS1984 |
| *var.curvicalcaratum* | Cameroon | Droissart & Simo 271 | BRLU | 10.77705;2.69501666666667 | WGS1984 |
| *var.curvicalcaratum* | Cameroon | Droissart & Simo 277 | BRLU | 10.8053333333333;2.70705 | WGS1984 |
| *var.curvicalcaratum* | Cameroon | Droissart 597 | BRLU | 10.5406111111111;2.75011666666667 | WGS1984 |
| *var.curvicalcaratum* | Cameroon | Droissart & Simo 622 | BRLU | 10.8053333333333;2.70705 | WGS1984 |
| *var.curvicalcaratum* | Cameroon | Droissart, Stevart & Simo 633 | BRLU | 10.5469833333333;2.74658333333333 | WGS1984 |
| *var.curvicalcaratum* | Cameroon | Droissart, Stevart & Simo 634 | BRLU | 12.5750833333333;3.29921666666667 | WGS1984 |
| *var.curvicalcaratum* | Cameroon | Annet 1376 | P | 10.4101583333333;3.07763333333333 | WGS1984 |
| *var.curvicalcaratum* | Cameroon | Breteler 1641 | P | 11.6166666667;3.55 | WGS1984 |
| *var.curvicalcaratum* | Cameroon | Breteler 1673 | K | 13.7333333333;4.5833333333 | WGS1984 |
| *var.curvicalcaratum* | Cameroon | Gregory 25375 | K | 9.23333333333333;4.16666666666667 | WGS1984 |
| *var.curvicalcaratum* | Cameroon | Jacques-Felix 4740 | K | 12.3666666666667;4.68333333333333 | WGS1984 |
| *var.curvicalcaratum* | Cameroon | Stevart & Pial 420 | K | 12.9555666666667;2.86821666666667 | WGS1984 |
| *var.curvicalcaratum* | Equatorial Guinea (Rio Muni) | Parmentier & Nguema 336 | BRLU | 10.9166666666667;1.83333333333333 | WGS1984 |
| *var.curvicalcaratum* | Equatorial Guinea (Rio Muni) | Stevart 763 | BRLU | 10.4666666666667;1.58333333333333 | WGS1984 |
| *var.curvicalcaratum* | Equatorial Guinea (Rio Muni) | Deman, Ndong, Bokung & Nguema 11 | BRLU | 10.9333333333333;1.78333333333333 | WGS1984 |
| *var.curvicalcaratum* | Equatorial Guinea (Rio Muni) | Parmentier 1681 | BRLU | 10.4666666666667;1.58333333333333 | WGS1984 |
| *var.curvicalcaratum* | Equatorial Guinea | Senterre, Obiang & Ngomo 2009 | ULB | 10.076;1.625 | WGS1984 |
| *var.curvicalcaratum* | Equatorial Guinea | Carvalho 5454 | MA | 9.78223888888889;1.80812777777778 | WGS1984 |
| *var.curvicalcaratum* | Gabon | Reitsma & Louis 1905 | WAG | 10.9166666666667;0.883333333333333 | WGS1984 |
| *var.curvicalcaratum* | Gabon | Halle 4964 | WAG | 10.9;0.98 | WGS1984 |
