# Supplementary material for: Phylogenetic climatic niche conservatism and evolution of climatic suitability in Neotropical Angraecinae (Vandeae, Orchidaceae) and their closest African relatives
Source: PeerJ. 2017 May 16;5:e3328. doi: 10.7717/peerj.3328 (PMC5436590; doi:10.7717/peerj.3328)
Supplement: Table S2 [file peerj-05-3328-s002.pdf]

	Comp.1	Comp.2	Comp.3	Comp.4	Comp.5	Comp.6	Comp.7	Comp.8	Comp.9	Comp.10
bio01	-0,0288495	0,3688074	0,0690193	-0,1142031	-0,0715514	-0,0571467	-0,0004193	-0,0244646	-0,02395	0,0098082
bio02	0,1951094	-0,0515607	-0,0594887	0,0611093	0,0117636	-0,1246334	-0,4751859	-0,2072857	-0,5147923	0,1548802
bio03	-0,172646	0,0465779	-0,0903358	0,1757897	-0,2260257	0,3401571	-0,1103309	-0,1320901	-0,3896132	-0,2539206
bio04	0,1723574	-0,1135727	0,2315657	-0,1632059	0,1808537	-0,2422877	-0,0519899	0,1533134	0,1399591	-0,0264529
bio05	0,0791478	0,3311953	0,1074218	-0,1456804	0,0454778	-0,1400139	-0,1484638	-0,0574941	-0,0881087	0,1688018
bio06	-0,13239	0,3046398	0,0310202	-0,0578209	-0,0884002	0,124011	0,1477648	0,0273936	0,0487598	-0,0361183
bio07	0,2104474	-0,0573393	0,0554539	-0,0579958	0,1343907	-0,2519152	-0,2848912	-0,0777411	-0,1265429	0,179844
bio08	0,0108641	0,3274675	0,1576828	-0,1956299	-0,092882	-0,1774383	0,0536513	0,0374684	-0,1456142	-0,1694821
bio09	-0,0821905	0,3508705	0,0120913	-0,0435232	-0,0423706	0,0904947	0,0306655	-0,0849335	0,0152849	0,0851749
bio10	0,0284161	0,3477886	0,1525816	-0,1730181	0,004513	-0,1266116	-0,0184488	0,027559	0,038066	0,0455274
bio11	-0,0832433	0,351987	-0,0173601	-0,042343	-0,1049591	0,0413669	0,0195998	-0,0701502	-0,0636307	0,0592202
bio12	-0,2236913	0,0463559	0,0199206	0,1387888	0,1067455	-0,1475777	-0,0544974	-0,0683811	-0,1127928	-0,0329547
bio13	-0,1798605	0,1113084	-0,1641603	0,1948733	0,2028675	-0,2443569	-0,0039075	0,066068	-0,0328575	-0,1102387
bio14	-0,1731745	-0,0340156	0,3448607	0,1066904	-0,0332021	-0,0292462	-0,2823657	-0,1895034	0,2316132	-0,0391341
bio15	0,1955186	0,1109638	-0,1572026	0,042682	0,0262167	-0,0933974	-0,214977	0,0242484	0,2485541	-0,6415159
bio16	-0,1874278	0,0977221	-0,1531324	0,1877775	0,2017586	-0,2454904	-0,0184737	0,0243338	-0,0816427	-0,0689486
bio17	-0,1858049	-0,0295608	0,3268278	0,1022309	-0,0326668	-0,0169703	-0,241588	-0,1682261	0,1598183	-0,0295298
bio18	-0,1712812	-0,0986254	0,0630849	0,0519433	-0,186909	-0,4176474	0,1465264	-0,0278615	-0,2433719	-0,2486137
bio19	-0,1685004	0,0881718	0,0806629	0,1030656	0,3091107	0,2230359	-0,2789516	0,3349216	-0,1559095	-0,1838765
bio20	0,2028358	0,0614794	0,1745162	0,258891	0,0764842	0,003369	0,130444	0,0507672	-0,0674516	-0,0174278
bio21	0,1902364	0,0315194	0,1999964	0,1583491	0,2634014	0,0165398	0,2262074	-0,1405001	-0,0887185	-0,1180721
bio22	0,175546	0,0940255	0,1066407	0,3556959	-0,159806	-0,0100742	-0,0180767	0,1947116	0,0300743	0,0088605
bio23	-0,0741739	-0,0712662	0,0012567	-0,3452139	0,4648696	0,0472884	0,1885688	-0,4736879	-0,107531	-0,2068634
bio24	0,184916	-0,007415	0,2950793	0,1322985	-0,0272671	-0,0912778	0,2064041	0,2708675	-0,1787119	-0,0470562
bio25	0,1318343	0,1443769	-0,0222642	0,3479688	0,1707229	0,1948004	0,115009	-0,3575784	0,0895647	-0,0590162
bio26	0,1835015	0,0588164	0,2272102	0,1392613	0,2747387	0,0259838	0,2041011	0,0280604	-0,0828122	0,0287982
bio27	0,16754	0,081692	-0,025895	0,3474295	-0,1992863	-0,0705529	0,0649396	-0,3055553	0,0385084	0,1407047
bio28	-0,2350423	-0,0156541	0,0269287	0,0823821	0,0592077	-0,0728484	0,0863648	-0,0414807	-0,0054585	0,1430809
bio29	-0,1926557	0,071268	-0,1993697	0,1792623	0,165101	-0,192408	0,0560985	0,0557183	0,136002	0,2056534
bio30	-0,1918024	-0,0722727	0,3140352	0,057305	-0,0459384	0,000767	-0,1096247	-0,1160749	0,1769599	0,0019417
bio31	0,1784698	0,1499411	-0,2373922	0,0749952	0,0507384	-0,1229168	-0,2333056	-0,0110858	0,2978234	-0,1490059
bio32	-0,2010911	0,0491004	-0,189203	0,1621933	0,1602897	-0,1824907	0,0692617	0,0279562	0,091091	0,24406
bio33	-0,2007131	-0,0668639	0,2923261	0,0487249	-0,0402597	0,0060812	-0,0649648	-0,0976446	0,1193147	0,0069019
bio34	-0,1877808	-0,1329024	0,0629973	0,0260711	-0,1989426	-0,2711385	0,1924172	-0,0063674	-0,1317753	-0,2232144
bio35	-0,1922417	0,0451752	0,1079227	0,0521504	0,265796	0,2372518	-0,1181005	0,3081085	-0,1155075	0,040394

	Comp.11	Comp.12	Comp.13	Comp.14	Comp.15	Comp.16	Comp.17	Comp.18	Comp.19	Comp.20
bio01	-0,0327612	-0,0002324	0,0477548	-0,0303561	-0,0039145	-0,0567873	-0,0704844	-0,0185654	0,0382373	-0,057724
bio02	0,0066965	-0,168072	-0,0052722	-0,1392181	0,0329968	0,0862498	-0,0186795	0,0962437	-0,0511274	-0,0309241
bio03	0,2722856	-0,1113004	-0,0068034	-0,1342505	-0,3551639	-0,2706413	0,1884234	-0,3076139	-0,0015277	0,009487
bio04	-0,1012323	0,1998068	-0,0671622	-0,1607748	-0,274712	-0,4052537	0,2622124	-0,2653876	0,0567511	0,037465
bio05	-0,0348654	-0,0812187	0,0122078	0,004148	0,1293253	-0,0691269	0,113558	-0,0252053	0,0463099	0,1223607
bio06	0,0486741	-0,0883935	-0,0418988	0,0856122	0,0817371	-0,0943612	0,0834308	0,0092426	-0,0102983	0,0923052
bio07	-0,082168	0,0290128	0,055923	-0,0900725	0,0182677	0,0455884	0,0033042	-0,0310593	0,0497646	0,0009309
bio08	0,0731481	0,3055968	0,1431037	-0,0974779	-0,3426394	0,1793795	-0,3484551	-0,0202667	-0,3597192	-0,0925426
bio09	-0,1568023	-0,1242875	-0,2326338	-0,0815502	0,0820286	0,0821803	0,2478211	0,0329222	0,1622729	0,0354822
bio10	-0,0345087	-0,0041898	0,0390023	-0,0404572	-0,0474786	-0,1921758	0,1208657	-0,064235	0,121297	-0,0286478
bio11	0,0235429	-0,1127645	0,0629185	0,0618585	0,1086423	0,0705844	-0,0806934	0,1204984	0,0398262	-0,0513983
bio12	0,0507075	0,2558325	-0,1488798	0,1741577	0,220389	0,1534063	-0,0204027	-0,4255074	0,257592	0,011401
bio13	0,245033	0,1336756	-0,2212734	-0,1434313	0,0750316	-0,1644361	0,1538171	0,4113267	0,0698903	0,0181275
bio14	0,0490506	-0,0834931	-0,1985093	0,3078859	-0,2326948	-0,003606	-0,1626705	0,147901	-0,1255432	-0,0189827
bio15	0,0997258	-0,0173983	0,0953699	-0,1273113	0,2463406	0,1526633	0,0969553	-0,1107766	-0,1344572	0,0004074
bio16	0,1552517	0,2513574	-0,1607987	-0,0436376	0,0407509	-0,013586	-0,0175999	0,0309114	-0,1134043	0,0265451
bio17	0,0421209	-0,0253605	-0,1587113	0,2187056	-0,0831443	0,0372074	-0,0294539	-0,0527826	0,117509	0,0176652
bio18	-0,3660794	-0,2086978	-0,1329048	0,2514587	0,1217691	-0,074254	0,1067762	-0,0019312	-0,2850248	0,2030254
bio19	-0,2247205	0,1559965	0,2188813	0,1904451	-0,0477215	-0,0509552	-0,1171812	0,1753551	0,2949303	-0,2187593
bio20	0,0066233	-0,0921338	0,0661289	0,0865207	0,0163694	-0,1006062	0,065363	0,0386516	-0,0252173	-0,1278286
bio21	0,0930031	-0,1499224	0,1245344	0,0865572	-0,0253534	-0,2978846	-0,0687208	0,0398793	0,0521971	0,2871115
bio22	-0,0003863	-0,0434511	-0,0104985	-0,2167063	0,1127288	-0,0915188	-0,5353617	-0,0461809	0,1898015	0,4669734
bio23	0,0960416	-0,0618112	0,1911898	0,0529683	-0,024971	0,105841	-0,1177509	0,0699631	0,1376119	0,2208113
bio24	0,211136	-0,0435196	-0,0012809	0,0633941	-0,2632647	0,5877337	0,3455099	0,0993125	0,1890093	0,1336901
bio25	-0,5438795	0,1641964	-0,2333713	-0,29199	-0,2124149	0,1764252	0,0481482	0,021543	-0,0578358	-0,0451044
bio26	0,1950608	-0,2618028	-0,2035772	0,0129402	0,2473658	-0,0458573	-0,1376362	-0,2093868	-0,2074113	-0,5482704
bio27	0,0464498	0,3778478	0,4869678	0,3137275	0,1061602	-0,1246267	0,2189781	0,06045	-0,0558401	-0,0795149
bio28	-0,0115244	0,0378448	0,1484715	-0,0899363	0,108827	0,182982	-0,0277762	-0,4425829	0,1079741	0,036661
bio29	0,1052085	-0,269838	0,1222063	-0,0895989	-0,2542562	-0,0342891	0,0305425	0,1292075	0,0241981	-0,0231702
bio30	0,1108669	-0,0649723	0,1946977	-0,3444613	0,1120702	0,0299184	0,0892819	0,165447	-0,160524	-0,0368846
bio31	-0,0442238	-0,3839345	0,1667419	0,1761027	-0,1613823	0,1031703	0,0489998	-0,1729265	0,1173445	-0,029346
bio32	0,0093139	-0,1362464	0,151987	-0,0128986	-0,265991	0,0582472	-0,1047493	-0,1910209	-0,1895765	0,0180245
bio33	0,1007671	-0,0312576	0,1905292	-0,3676942	0,1890847	0,0581728	0,129043	-0,0167127	0,0252161	0,0170774
bio34	-0,2938914	-0,1779682	0,2419406	-0,1812076	-0,0325181	-0,0707498	-0,106523	0,0818539	0,3365664	-0,344071

bio35	-0,2579769	-0,079938	0,2481196	0,031761	0,1298634	0,032816	0,1767543	-0,0580323	-0,4132933	0,2258296
	Comp.21	Comp.22	Comp.23	Comp.24	Comp.25	Comp.26	Comp.27	Comp.28	Comp.29	Comp.30
bio01	0,0373637	0,044066	0,0138992	0,1934835	0,0699704	0,1238587	-0,154036	0,100372	0,1066011	0,1763336
bio02	-0,0313619	-0,1433828	-0,023457	0,0169763	-0,1089585	0,253594	-0,0642102	-0,376366	0,2123186	0,1321561
bio03	0,0834269	0,0769499	0,079167	-0,0308517	0,0511087	-0,1446461	-0,0018515	0,1696285	-0,0856258	-0,0458689
bio04	0,1122017	-0,0473324	0,1399644	-0,0496702	-0,1178017	0,1089825	-0,0501688	-0,1721849	0,0875666	0,0264973
bio05	-0,0759791	0,0083972	-0,0409228	-0,2589026	0,2413131	-0,4747493	0,1898645	-0,155241	-0,2120714	-0,0512294
bio06	-0,0578231	-0,0133932	-0,0252469	-0,1721172	0,063598	-0,1332514	0,1238833	-0,5376163	0,0306322	0,0882567
bio07	-2,44E-05	0,0216144	-0,006455	-0,0273035	0,1312204	-0,2492748	0,0225766	0,4581785	-0,2098373	-0,1390173
bio08	0,0545024	-0,0225541	-0,2723791	-0,049484	-0,1723609	0,0904545	0,0887866	-0,0367976	-0,2065478	-0,0719629
bio09	-0,0835661	-0,3117383	0,1909165	-0,1613586	-0,456603	0,2984874	-0,0175395	0,1917325	-0,3474948	-0,0793654
bio10	0,0709929	0,1002608	0,128199	0,1823659	0,1093189	0,0679208	-0,1334947	0,105466	0,3414646	0,0190974
bio11	-0,0183482	0,071949	0,0562737	0,1703045	0,1571174	0,0311403	-0,1066547	0,2184924	0,3022106	-0,0800401
bio12	-0,009899	0,1070234	-0,1514082	0,1114072	-0,0607982	0,027765	-0,2025002	-0,1813567	0,0256447	-0,5061166
bio13	0,294594	-0,0563345	-0,2353998	0,1416017	-0,0556579	-0,1160376	-0,0458428	0,0706688	-0,1173021	0,2220564
bio14	0,0023232	-0,219576	0,171206	0,1189126	-0,0514104	-0,285971	-0,3162418	-0,0701978	-0,0293101	0,0791572
bio15	-0,045605	-0,3328642	0,0562061	-0,2043338	0,1908656	0,0240548	-0,1307961	0,052507	0,1254926	-0,0501312
bio16	-0,3446513	0,1127734	0,3908749	0,2214935	-0,0143641	-0,0021384	0,3293214	-0,0370297	0,0076822	0,0554418
bio17	0,1250083	-0,151556	-0,1577602	-0,0597066	0,1928444	0,2856267	0,501434	0,1252937	0,0862124	0,0015781
bio18	0,0714227	0,2940913	-0,012582	-0,2310909	-0,0143582	0,0932465	-0,0946545	0,1199754	0,0161583	0,1009106
bio19	-0,1161463	0,187146	0,0288606	-0,3542485	-0,0431961	0,0475632	-0,0841768	0,0413535	-0,0014813	0,1471204
bio20	-0,0120487	0,0077342	0,040752	0,215292	0,5013798	0,3702635	-0,1620302	-0,1553164	-0,5234621	-0,0295177
bio21	-0,5025393	-0,155425	-0,3803222	0,0667398	-0,1840841	-0,0182428	-0,0204526	0,1373713	0,1377389	-0,0393097
bio22	0,2645289	0,0381958	0,2436377	-0,0687854	-0,0820658	-0,0019887	0,0257892	-0,0156374	-0,0206445	-0,0155429
bio23	0,2926206	0,0580959	0,2866555	-0,0316733	0,0255118	0,0624154	0,009445	-0,0582933	-0,0797537	0,0116122
bio24	0,0032821	0,0204049	0,1186684	-0,0119845	-0,0098258	-0,1097832	-0,0003585	0,0014134	0,1406433	0,0374596
bio25	0,032009	0,1157241	-0,0881638	0,0049504	0,1116098	-0,1282863	0,0070288	-0,05919	0,1351693	0,0122515
bio26	0,1978754	0,0517437	0,0967591	-0,1419282	-0,1901009	-0,1126001	0,1036335	0,0645131	0,1036776	0,0316344
bio27	0,1836598	-0,0661804	0,1350996	-0,1137912	-0,1864121	-0,0253936	0,0615547	0,0091876	0,0497931	0,0194088
bio28	0,0312964	-0,1871915	-0,1846291	0,0207371	0,0073681	-0,1021584	-0,2557132	0,0232485	-0,1112916	0,5688518
bio29	0,2495238	-0,0507327	-0,2151234	-0,2739853	0,0568396	0,0882858	-0,1257349	-0,0567621	0,1222315	-0,3344897
bio30	-0,1881165	0,3062401	0,1190499	-0,016833	-0,165697	-0,0449889	-0,2708508	-0,0817815	-0,1136653	-0,184198
bio31	0,0297197	0,3693167	-0,101406	0,2911941	-0,2994777	-0,0314679	0,1698586	-0,0912272	-0,1727066	0,1116054
bio32	-0,2658879	-0,1157757	0,2804615	-0,1879338	0,1478256	0,0974031	0,0220815	0,0673651	0,0509334	0,0570624
bio33	-0,0196926	0,2069247	-0,1446081	-0,0574485	0,0498595	0,1698645	0,256459	0,0393812	0,0575933	0,1383368
bio34	-0,0284468	-0,317212	0,0977436	0,2057341	-0,0237495	-0,2014136	0,186271	-0,10862	-0,0132504	-0,1297104
bio35	0,2408994	-0,2242949	0,0090351	0,3457094	-0,0752112	-0,083405	0,1334471	0,0049819	0,0357452	-0,15716
	Comp.31	Comp.32	Comp.33	Comp.34	Comp.35					
bio01	-0,0452829	0,8073264	-0,1613863	-0,027053	5,79E-09					
bio02	-0,0310979	-0,0330412	0,0013887	-0,014364	4,75E-09					
bio03	0,0085	0,0097883	0,0058838	0,0037768	-3,22E-09					
bio04	-0,0420139	-0,0031319	-0,0661895	0,4072572	-5,14E-09					
bio05	-0,0172175	0,0297822	-0,0088017	0,0308263	0,4896444					
bio06	-0,0505175	-0,0120194	-0,0095509	0,0163888	-0,6431401					
bio07	0,0408656	0,0378992	0,0031132	0,0077345	-0,5887437					
bio08	0,0226948	-0,1459639	0,0138017	0,0004683	4,76E-09					
bio09	0,0812482	-0,0548356	-0,0108273	-0,0037646	-2,78E-09					
bio10	0,0453328	-0,3599044	0,1971671	-0,5734196	1,06E-08					
bio11	-0,0524157	-0,3041463	-0,0329973	0,6796032	-4,38E-09					
bio12	-0,0494714	0,0045083	-0,2131706	-0,0545382	-3,95E-09					
bio13	-0,2890417	-0,1129578	-0,2096565	-0,0356552	5,56E-09					
bio14	0,2920266	-0,044627	-0,1674054	-0,0110087	-1,16E-09					
bio15	0,0146494	0,0207802	0,0209627	-0,0093624	9,55E-10					
bio16	0,3016342	0,0961471	0,3148482	0,0754805	-1,19E-09					
bio17	-0,3195542	0,0633623	0,254235	0,0153437	3,64E-09					
bio18	0,0277719	-0,0040747	0,016539	0,0052594	-1,77E-09					
bio19	0,0179488	-0,0175188	0,0033841	0,0069682	-1,06E-09					
bio20	0,0652832	-0,0825048	0,0127606	0,0241032	-5,32E-09					
bio21	0,0046892	0,0202603	0,0071851	-0,0089375	5,94E-09					
bio22	-0,0178141	-0,022265	0,0053933	-0,0053406	-2,19E-09					
bio23	-0,0113955	0,0018445	-0,0092003	0,0017827	-3,07E-09					
bio24	-0,0320261	0,0666464	-0,0211343	0,0023937	-2,85E-09					
bio25	-0,0217008	-0,0018575	0,0049023	-0,0039809	-2,60E-09					
bio26	-0,0203393	0,0189372	-0,007217	-0,0055176	3,31E-09					
bio27	0,0043127	0,0194465	-0,0116189	-0,0045207	2,26E-09					
bio28	0,037999	-0,0762617	0,333232	0,1028963	1,36E-09					
bio29	0,3436393	0,1576927	0,2735393	0,0484197	-6,64E-09					
bio30	-0,4020804	0,0682995	0,2856714	0,0148289	-5,38E-09					
bio31	-0,031653	-0,0540086	-0,0283829	0,0391562	-4,94E-10					
bio32	-0,35815	-0,0970672	-0,41251	-0,1211291	5,86E-09					

bio33	0,4407168	-0,0877208	-0,4552621	-0,0191752	5,65E-09					
bio34	-0,03809	0,0012993	-0,0196692	-0,0105187	-1,87E-09					
bio35	-0,0146127	0,0338031	-0,0096816	-0,0126911	2,28E-09					
