# Supplementary material for: Phylogenetic climatic niche conservatism and evolution of climatic suitability in Neotropical Angraecinae (Vandeae, Orchidaceae) and their closest African relatives
Source: PeerJ. 2017 May 16;5:e3328. doi: 10.7717/peerj.3328 (PMC5436590; doi:10.7717/peerj.3328)
Supplement: Table S3 [file peerj-05-3328-s003.pdf]

**Species**	**ITS**	**trnL-F**
A. chevalieri	AF5063201	AF506339.1
A. cultriforme	AF5063211	AF506340.1
A. eichlerianum	AF5063221	AF506341.1
A. erectum	DQ0915661	DQ091447.1
C. fasciola	AF5062951	AY147226.1
C. lansbergii	AF5062971	AF506324.1
C. micranthum	AY1472201	AY147227.1
C. pachyrrhizum	AF5063011	AF506327.1
C. poeppigii	AF5063021	AF506329.1
C. tyrridion	AF5063051	DQ091446.1
D. barrettiae	AF5063081	AF506330.1
D. fawcetti	AF5063091	AF506331.1
D. lindenii	AF5063181	AF506338.1
D. porrectus	JN1760941	AF506335.1
D. sallei	AY1472251	AY147234.1
D. varius	AY1472221	AY147230.1
P. pyramidalis	KF6722121	KF662339.1
